# Environment‐Adaptive Coassembly/Self‐Sorting and Stimulus‐Responsiveness Transfer Based on Cholesterol Building Blocks

**DOI:** 10.1002/advs.201700552

**Published:** 2017-11-08

**Authors:** Pengyao Xing, Huijun Phoebe Tham, Peizhou Li, Hongzhong Chen, Huijing Xiang, Yanli Zhao

**Affiliations:** ^1^ Division of Chemistry and Biological Chemistry School of Physical and Mathematical Sciences Nanyang Technological University 21 Nanyang Link 637371 Singapore Singapore; ^2^ School of Materials Science and Engineering Nanyang Technological University 50 Nanyang Avenue 639798 Singapore Singapore

**Keywords:** cholesterol, coassembly, nanostructures, property transfer, stimulus responsiveness

## Abstract

Manipulating the property transfer in nanosystems is a challenging task since it requires switchable molecular packing such as separate aggregation (self‐sorting) or synergistic aggregation (coassembly). Herein, a unique manipulation of self‐sorting/coassembly aggregation and the observation of switchable stimulus‐responsiveness transfer in a two component self‐assembly system are reported. Two building blocks bearing the same cholesterol group give versatile topological structures in polar and nonpolar solvents. One building block (cholesterol conjugated cynanostilbene, CCS) consists of cholesterol conjugated with a cynanostilbene unit, and the other one (C_10_CN) is comprised of cholesterol connected with a naphthalimide group having a flexible long alkyl chain. Their assemblies including gel, crystalline plates, and vesicles are obtained. In gel and crystalline plate phases, the self‐sorting behavior dominates, while synergistic coassembly occurs in vesicle phase. Since CCS having the cyanostilbene group can respond to the light irradiation, it undergoes light‐induced chiral amplification. C_10_CN is thermally responsive, whereby its supramolecular chirality is inversed upon heating. In coassembled vesicles, it is interestingly observed that their responsiveness can be transferred by each other, i.e., the C_10_CN segment is sensitive to the light irradiation, while CCS is thermoresponsive. This unprecedented behavior of the property transfer may shine a light to the precise fabrication of smart materials.

## 1 Introduction

Biological species such as phospholipid cell wall and DNA are formed by versatile noncovalent aggregation of multiple components, where small molecules or macromolecules are held together individually or cooperatively, forming biomaterials with diverse functions. Unlike genetically encoded biomolecule organization, abiotic self‐assembly of a multiple‐component system often shows complex routes, whereby the self‐assembly pathway complexity is largely determined by ambient environment, intercomponent interaction, and structural similarity of components.^1^, ^2^, ^3^, ^4^ Normally, three scenarios can be expected when the self‐assembly is triggered in a multicomponent system, i.e., self‐sorting, synergistic assembly, and heterojunction.^5^ Self‐sorting describes selective recognition of mutual counterparts to give orthogonal or parallel aggregation,[[qv: 6a]] which could be further classified as narcissistic and social self‐sorting.[[qv: 6b]] Thus, self‐sorting is able to endow systems with abundant aggregates, which enables the fabrication of highly ordered materials with sophisticated structure and functionality.^7^ Alternatively, when the interaction between different components is relatively strong, they may give rise to mixed supramolecular arrangement either randomly or specifically.^8^ The third outcome of multicomponent assembly, often accompanied by heterojunction structural formation, could be assigned as a top–down method, because it needs the preassembly of one component as a seed, followed by the self‐assembly with another component to give suprastructures.^9^

Designing synergistic self‐aggregation or coassembly from multiple components is of vital importance in supramolecular chemistry and materials science.^10^ Coassembly allows materials integration at a molecular scale, showing specific advantages over physical blend or hybrid at micro/nanoscale prepared by self‐sorting.^11^ For instance, liposome and polymersome, which are vesicles self‐assembled from lipids and polymeric amphiphiles, are often integrated with targeting ligands, drugs, and bioimaging agents as payloads. Payload loading will be more effective if the coassembly pathway is adopted.^12^ In addition, the coassembly of building blocks appended with electron‐rich and electron‐poor fused aromatic rings could significantly change the luminescence property and improve electron mobility.^13^ The coassembly can occur in any dimensional structures, and sometimes alter pristine morphology and dimension by changing molecular packing parameters. For example, Yan and coworkers observed that the 1D morphology of phenylalanine dipeptide could be finely tuned through the coassembly with dianionic porphyrin, and the generated spheres exhibit excellent photocatalysis and photodynamic properties.^14^, ^15^, ^16^

A favorable strategy to construct a coassembly system is to reinforce intercomponent interactions that typically are metal–ligand coordination, hydrogen bonding, halogen bonding, π–π stacking, and van der Waals interactions.^17^, ^18^, ^19^ Apart from enhancing interactions, another common design protocol is to increase structural similarity of different components.^20^, ^21^, ^22^ Structural similarity reduces the discrimination and exclusionary possibility during the aggregation process, resulting in the interdigitation arrangement. We recently fabricated coassembled vesicular particles utilizing two aromatic glutamate‐based amphiphiles with considerable structural similarity to achieve controllable luminescent color conversion, and the π–π stacking interaction between two components was evidenced as the dominant driving force for the coassembly.^23^ In spite of these developments, rational design of coassembly systems still remains a challenge.^24^ Tiny variation of functional groups would have profound influence on preferred molecular arrangement in aggregates, where the competition with self‐sorting is negligible.

In coassembly arrays, different components are capable of impacting each other, as they are confined in aggregates with a high affinity (distance between building units might be less than 10 Å). These influences can be brought by charge transfer (CT) between electron‐poor and rich moieties, energy transfer (ET) between moieties with well‐overlapped spectra, and chirality transfer.^25^, ^26^, ^27^, ^28^, ^29^, ^30^ For instance, researchers[[qv: 25b,c]] utilized building blocks bearing naphthalene diimide and naphthalene moieties to flexibly control the coassembly/self‐sorting behavior. Schenning and coworkers^30^ demonstrated the control over ET between naphthalene and benzothiadiazole fluorine domains of amphiphilic components in random mixing or self‐sorting self‐assemblies. It should be noted that the chirality transfer stands for the acquisition of supramolecular handedness for a component without chiral amplification capability from another component with supramolecular chirality.^31^ Regardless of these phenomena, transferring properties from one building block to another is still very difficult to realize. It is well known that a considerable number of organic self‐assemblies belong to nonequilibrium processes, which endow the self‐assembled systems with stimuli‐responsiveness. Study of stimulus‐responsiveness transfer in multicomponent noncovalently bonded system helps better understand the synergistic effect in living cells, and the responding to external stimuli and transference to adjacent unit are reminiscent of intracellular information conduction. While achieving the transfer of stimuli responsiveness within coassemblies is of great significance to the better understanding of biological information conduction and the fabrication of smart functional materials, it has not been well investigated to the best of our knowledge. Although responsive species have been combined to give multicomponent arrays, the responsiveness is still limited to the intrinsic species without the observation of transference to other component,[[qv: 29b]] showing independent behaviors in either self‐sorting or coassembled states.

**Figure 1:**
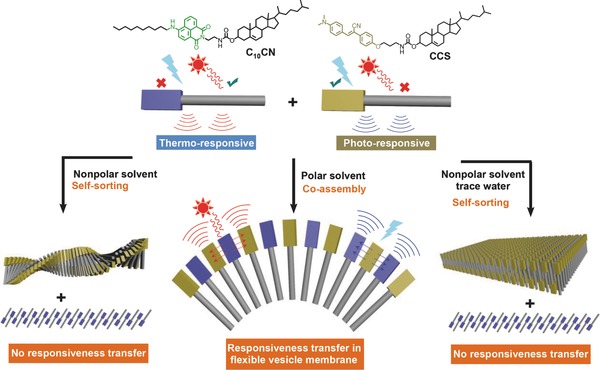
Chemical structures and schematic models of C_10_CN and CCS, diverse self‐assembled modalities in polar/nonpolar solvents, and coassembly/self‐sorting behavior of C_10_CN/CCS mixture. In this representation, the lighten symbol stands for the UV light irradiation; the red sun represents heating stimulus; red and blue lines stand for the responsiveness of building blocks to thermo‐ and photo irradiation stimuli.

To this end, two new small organic building blocks (**Figure** **1**) with specific structural similarity were rationally designed and synthesized (Figures S1–S6, Supporting Information), namely cholesterol conjugated cynanostilbene (CCS), and cholesterol connected naphthalimide having a flexible long alkyl chain (C_10_CN). Several factors were taken into account in this design. (1) The employment of C_10_CN with flexible long alkyl chain and π‐conjugated naphthalimide group may favor geometrical diversity in response to the solvent environment and temperature. This may help us optimize ambient condition for the coassembly.^32^ (2) CCS appended with cynanostilbene could respond to UV light irradiation.^33^ (3) C_10_CN and CCS share the same cholesterol and spacer groups and have different polar head groups, providing possibilities for both coassembly and self‐sorting. By systematic studies, it was found that C_10_CN self‐assembles into gels, crystalline flowers, and vesicles in nonpolar solvents, nonpolar solvent with a trace amount of water, and aqueous solution, respectively (Figure 1). C_10_CN and CCS self‐sorted in gel and crystalline phases in nonpolar solvents but coassembled in the vesicle phase in water. Further studies revealed that the photoresponsiveness of CCS and the thermoresponsiveness of C_10_CN could be transferred to each other. Specifically, the light irradiation induces the chiral amplification of the CCS self‐assembly, while C_10_CN is the light‐insensitive species with chiral amplification in the C_10_CN/CCS coassembly. Upon heating, C_10_CN vesicles undergo the chirality inversion on account of the dehydration, while thermoinsensitive CCS presents thermoresponse in the C_10_CN/CCS coassembly.

## 2 Results and Discussion

### 2.1 Individual Assemblies

**Figure 2:**
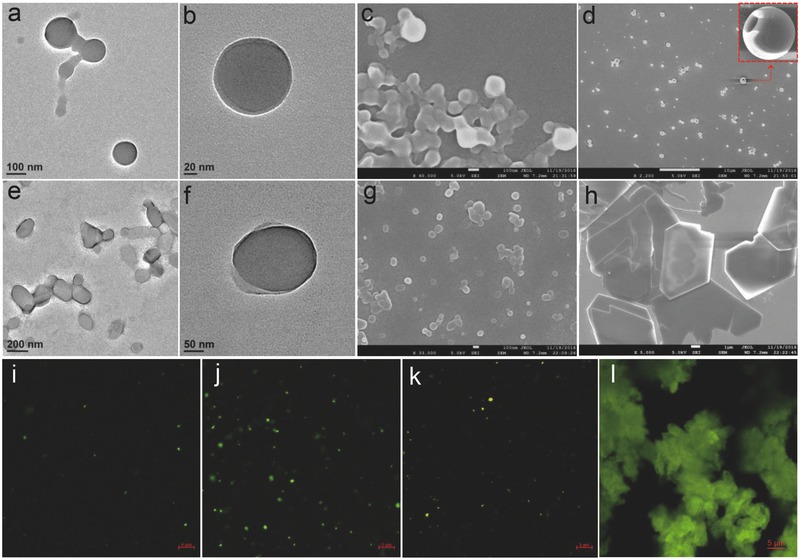
Self‐assembly of CCS and C_10_CN in THF–water mixture. a–c) TEM and SEM images of CCS vesicles (*f*_w_ = 90 vol%, *c* = 10^−4^ m). d) SEM image of vesicles formed by CCS at a low water volume fraction (*f*_w_ = 60 vol%, *c* = 10^−4^ m). e–g) TEM and SEM images of C_10_CN vesicles (*f*_w_ = 90 vol%, *c* = 10^−4^ m). h) SEM image of aggregates formed by C_10_CN at a low water volume fraction (*f*_w_ = 60 vol%, *c* = 10^−4^ m). i–l) CLSM images of CCS aggregates (*f*_w_ = 90 vol%, *c* = 10^−4^ m), CCS aggregates (*f*_w_ = 70 vol%, *c* = 10^−4^ m), C_10_CN aggregates (*f*_w_ = 90 vol%, *c* = 10^−4^ m), and C_10_CN aggregates (*f*_w_ = 70 vol%, *c* = 10^−4^ m), respectively.

Both of CCS and C_10_CN exhibit significant adaption toward solvent environment by giving diversified aggregated structures. Solvent exchange protocol enables the well‐defined self‐assembly of organic building blocks with minimal hydrophilicity, by which means CCS and C_10_CN with flexible alkyl chains and polar aromatic heads are capable of forming aggregates in aqueous media.^34^ To facilitate the self‐assembly, CCS and C_10_CN were predissolved in high water‐miscible solvent tetrahydrofuran (THF), which were then injected into water under the sonication. Colloidal stabilities of the assemblies show considerable dependence on the solvent ratio or water volume fraction (*f*_w_). CCS dispersion was quite stable without any precipitation (*f*_w_ from 60 to 90 vol%), yet C_10_CN dispersion started to precipitate upon the incubation time when *f*_w_ was lower than 80 vol%, which might be due to the long alkyl chain of C_10_CN. Then, the morphologies of different dispersions were examined by electron microscopic techniques including scanning electron microscope (SEM) and transmission electron microscope (TEM) shown in **Figure** **2** and Figures S7 and S8 (Supporting Information). In high *f*_w_ condition (90 vol%), CCS gave rise to spherical assemblies with diameter about 100 nm (Figure 2a). Magnified TEM images display clear periphery (skin/pool structure), suggesting their hollow nature (Figure 2b), i.e., the vesicle formation.^35^ Stepwise decrease of *f*_w_ influences the size rather than the basic morphology. By decreasing *f*_w_ from 90 vol% to 60 vol%, the diameter of aggregates elevated from 100 to ≈500 nm without varying the vesicular structure. In the SEM studies of CCS, drying process on silicon wafer may result in some broken vesicles as observed (Figure 2d; Figure S7f, Supporting Information). Holes on the surface of these nanoparticles well elucidate the presence of hollow interiors.^36^ Similarly, C_10_CN dispersion with *f*_w_ = 90 vol% contains vesicles with slight aspect ratio. As compared to CCS, C_10_CN appended with a long alkyl chain tends to form membranes with larger curvature, and thus may mildly improve 1D growth. When lowering the *f*_w_ value from 90 to 60 vol%, gradual disappearance of vesicles and the appearance of microscale crystalline plates could be observed (Figure S8e–h, Supporting Information). A possible reason is that less amount of poor solvent water might provide a favorable environment for thermodynamic aggregation to form crystalline structures. Confocal laser scanning microscopy (CLSM) was used to confirm the morphologies in wet state (Figure 2i–l). After being excited by laser (405 nm), both of CCS and C_10_CN aggregates show greenish yellow emission. Highly dispersed dots were found in vesicle samples. The morphology of crystalline plates under CLSM was also consistent with SEM and TEM observations. In good agreement with electron microscopic studies, lower *f*_w_ (70 vol%) generates dense and larger aggregates than that of high *f*_w_. Solvent ratio‐dependent absorption and emission spectra (Figure S9a–d, Supporting Information) reveal that both of CCS and C_10_CN formed J‐type π–π stacking arrays with aggregation‐induced emission (AIE)^37^ and aggregation caused quenching (ACQ) effects in polar solvent environment, respectively. Concentration‐dependent absorption and emission studies show the critical aggregation concentration of 10^−5^ and 2 × 10^−5^ m for CCS and C_10_CN, respectively (Figures S10 and S11, Supporting Information).

Then, the self‐assembly behavior was examined in nonpolar solvents such as hexane and decane. The dispersion of CCS in these solvents only resulted in crystalline precipitation. For CCS in nonpolar solvents, molecular aggregation favors 3D growth with large aggregation number, enabling the emergence of bulk crystalline aggregates. Nevertheless, though C_10_CN shares the cholesterol and spacer groups with CCS, the long alkyl chain significantly facilitates its 1D growth in nonpolar solvents. Both the solvent exchange and direct dispersion methods gave rise to high quality organogels in decane or hexane. Macroscopically, these gels are highly transparent, thermoreversible, thixotropic, and highly stable in sealed vials for at least 3 months. Notably, the critical gelation concentration is as low as 1 × 10^−3^ m (≈0.08% w/v), indicating their high solvent content and supergelation nature.^38^

**Figure 3:**
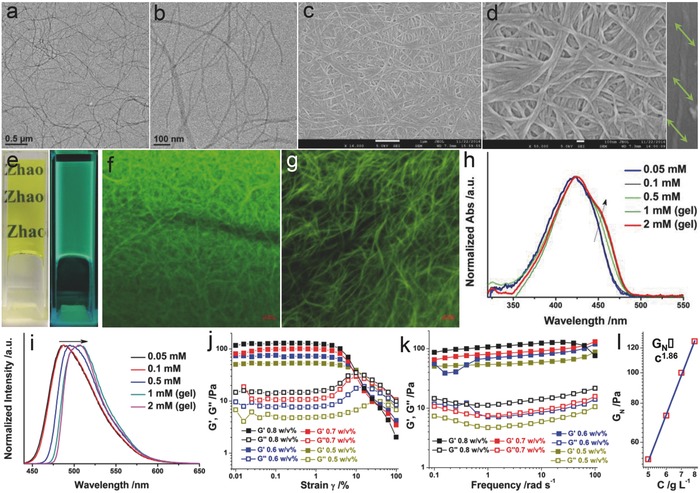
Self‐assembly of C_10_CN in nonpolar solvents. a–d) TEM and SEM images of nanofiber formation in decane with 10 vol% THF. Inset of (d) is an example of twisted individual nanofiber adopting M‐type handedness. e) Digital images of macroscopic gels formed in decane under natural and UV light (365 nm). f,g) CLSM images of gels formed in decane and hexane, respectively. h,i) Concentration‐dependent UV–vis absorption and emission spectra, respectively. j,k) Dynamic strain sweep and dynamic frequency sweep of decane gels. (l) Complex modulus as a function of concentration with double logarithmic axis.

Good properties of C_10_CN organogels originate from the nanostructures. We first investigated the nanostructures of organogels by TEM and SEM (**Figure** **3**a–d). Long, flexible, and infinitely aggregated nanofibers constitute the gel networks. Ascribed to intrinsic molecular chirality of the cholesterol unit, some nanofibers present supramolecular chirality, whereby left‐handed (M‐) twisted fibers could be found. By counting 50 individual nanofibers, the mean diameter of gel fibers was determined as 11 nm. This result means that there was almost no extension on other dimensions during the aggregation, which is reminiscent of supramolecular polymeric gels.^39^ We also observed considerably thick bundled fibers in Figure 3c and Figure S12 (Supporting Information).

C_10_CN organogels showed strong green fluorescent emission under a UV lamp, of which morphology could be probed via CLSM. As shown in Figure 3f,g, fibrous networks are highly entangled, suggesting that the solvent evaporation barely has an influence on gel morphologies. It should be noted that the bundle effect in hexane might be more active due to the presence of thicker fibers than that in decane. Upon increasing the concentration, C_10_CN molecule transforms from monomeric state into aggregated state, which is directed by hydrogen bonding between imide and amide groups. Gelation induces the emergence of a small shoulder absorption peak located at 450 nm (Figure 3h) and red‐shifted emission from 486 to 507 nm (Figure 3i), indicative of π–π stacking or hydrogen bonding formation by naphthalimide unit. The gradual increase of the decane content in THF–decane mixture could also generate organogels, showing a blue shift in emission spectra (Figure S13, Supporting Information). We then examined the absorption/emission properties of C_10_CN in solvents with different polarities under the same concentration (Figure S14, Supporting Information), and found that the absorption/emission wavelengths are ultrasensitive to solvent polarities.

Oscillatory rheology experiments were performed to investigate the viscoelastic properties of gels (Figure 3j,k). In dynamic strain sweep (frequency = 1 rad s^−1^), gels showed long linear viscoelastic region from 0.01% to 10%. At the condition of γ = 0.1%, both of storage modulus (*G*′, representing elasticity) and loss modulus (*G*″, representing viscosity) exhibit an independence to the applied frequency (0.1–100 rad s^−1^). At the meantime, *G*′ values are almost 1 order higher than that of *G*″, and thus the gel system behaves as solid rather than liquid. Mechanical strength of gels is concentration dependent. Complex plateau modulus, which is the weighted average of *G*′ and *G*″, has a good linear correlation with concentration (g L^−1^) on a double logarithmic scale having a slope of 1.86 (Figure 3l). The relationship *G*_N_ ∝ *C* ^1.86^ was then obtained. This relationship implies that this organogel system is close to the Cates living polymerization model (*G*_N_ ∝ *C* ^2.0^), demonstrating the existence of supramolecular polymerization‐like process during the gelation in nonpolar solvents.^40^ Surprisingly, we found that a trace amount of water from air that was absorbed in THF stock solution hindered the gelation by forming precipitates (with micro/nanoflower morphology; Figure S15, Supporting Information). Gel collapses even when 0.03 vol% or more water exists, reflecting highly environmental adaptive properties of C_10_CN.

**Figure 4:**
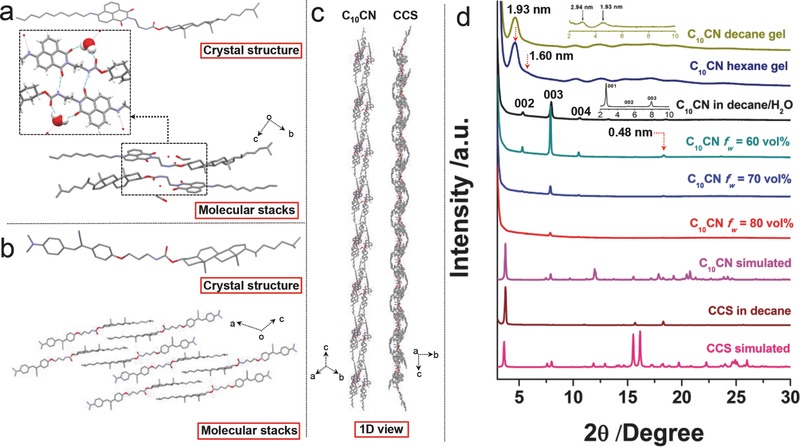
a) X‐ray single crystal structure and stacking of C_10_CN, inset of which highlights the hydrogen bonding formed by water molecule and C_10_CN. b) X‐ray single crystal structure and stacking of CCS. Hydrogen atoms are omitted for clarity. c) 1D views of C_10_CN and CCS molecular stacks to exhibit helical topologies along *c* axis. d) Powder XRD patterns of different samples, insets of which show corresponding small angle XRD profiles. More single crystal information could be found in Tables S1 and S2 (Supporting Information).

In order to probe the contribution of water molecule to the phase behavior, we analyzed the single crystal structure of C_10_CN. High quality crystals of C_10_CN were obtained in dichloromethane/ethyl alcohol mixed solvent system in the presence of a small amount of water. Single crystal structure and molecular stacking in a unit cell are displayed in **Figure** **4**a. No effective π–π stacking between naphthalimide groups was found in the stacking structure. Solvent molecules including water and ethanol participate in the crystallization via hydrogen bonds. From the highlighted inset in Figure 4a, it could be seen that, two C═O groups from amide and imide units are linked by one water molecule via hydrogen bonding interactions, of which distances are 1.83 and 2.17 Å, respectively. Meanwhile, another imide C═O group was hydrogen‐bonded (2.2 Å) to the amide N─H group. Thus, water molecule behaves as the linker of C_10_CN stacks, which has a high possibility in improving the molecular stacking along 3D (crystalline flowers) rather than 1D (fibers). This speculation was also confirmed by Sureshan and coworkers who employed a cyclohexane‐1a,3a‐diol motif to study the effect of water on the gelation crystallization balance.^41^ We also utilized Fourier transform infrared spectroscopy to verify hydrogen bonding variations in gels and crystalline flower structures (Figure S16, Supporting Information).

For comparison, we also analyzed the single crystal structure of CCS obtained in dichloromethane (DCM)/hexane (Figure 4b). In the crystal state, no hydrogen bonding can be observed. Although it exhibits a relatively plain aromatic surface with a torsion angle of 177. 94°, the nearest distance between cyanostilbene groups is larger than 5 Å, reflecting the absence of π–π stacking interaction. However, there is strong van der Waals interaction between cholesterol groups, which stack closely to each other with quite short distance (2–3 Å). Due to the intrinsic molecular chirality of cholesterol, the stacking of C_10_CN and CCS adopts a slipped manner. Along the *c* axis, the 1D molecular arrangement exhibits zigzag conformation (Figure 4c), suggesting that the crystal superstructure may be chiral on account of particular molecular stacking, which also supports the helical orientation observed in high resolution TEM (HRTEM) image (Figure S15e, Supporting Information). Additionally, the aggregates including crystals, flower structures, organogels, and vesicles were subjected to film‐based powder X‐ray diffraction (XRD) characterization to gain more structural information (Figure 4d). Except for the vesicle samples that gave amorphous structures, other samples showed diffraction peaks respectively, implying the existence of long‐range ordered molecular arrangements. The powder XRD pattern of CCS‐based crystalline precipitates formed in decane was in good agreement with simulated pattern from its single crystal data. For C_10_CN, peak locations of crystalline precipitates or gels varied significantly as compared to the simulated one, indicating that, under three different self‐organization scenarios, C_10_CN adopts distinct molecular arrangements. For the crystalline precipitates induced by water, similar diffraction peaks located at 5.4°, 8.0°, 10.7°, and 13.2° corresponding to the *d*‐space values of 1.64, 1.1, 0.83, and 0.67 nm possess ratios of (1/1):(1/1.49):(1/1.98):(1/2.45), revealing the lamellar molecular stacking of (1/2):(1/3):(1/4):(1/5).^42^ The peak at 18.5° with *d*‐spacing of 0.48 nm further verifies the distance found from HRTEM observations. Hump peaks appeared in powder XRD patterns of organogels on account of 1D characteristics, and the first two order peaks corresponding to the *d*‐spacing of 1.93 and 1.60 nm, are close to the ratio of √4:√3, suggesting that self‐assembled units may take cubic arrangements. The estimated molecular arrangements in various aggregates were also supported by the small‐angle XRD profiles displayed in insets of Figure 4d.

### 2.2 Coassembly and Self‐Sorting Behavior of C_10_CN and CCS

**Figure 5:**
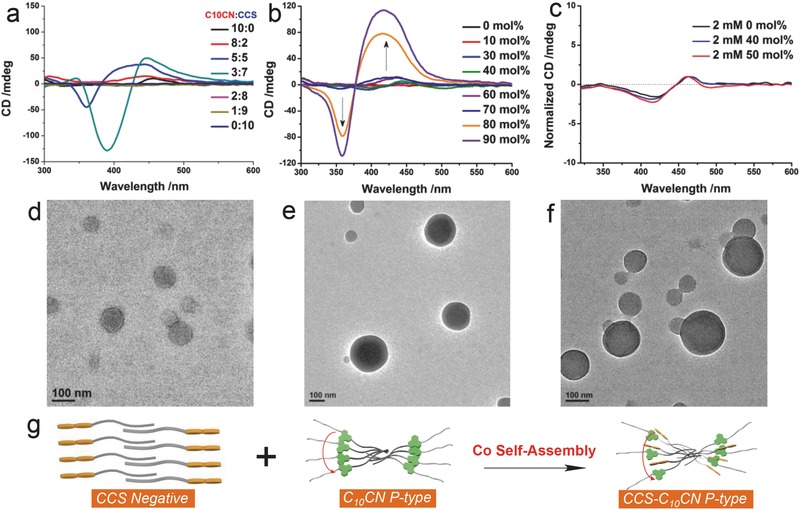
Coassembly and self‐sorting behavior of CCS and C_10_CN. a) CD spectra of CCS–C_10_CN coassembled system with different molar ratios. b) CD spectra of CCS upon the titration of C_10_CN (concentration of CCS: 10^−4^ m). c) Normalized CD spectra with the addition of CCS in decane gel of CD spectra of C_10_CN. d–f) TEM images of coassembly systems with the molar ratios of 2:8, 5:5, and 8:2 (CCS:C_10_CN, total concentration: 10^−4^ m). g) Schematic representation of coassembly induced chiral amplification, where CCS and C10CN were cartooned as yellow and green species, respectively. Samples for vesicles were all prepared in THF–water mixture (*f*_w_ = 90%). Note that all emission spectra were excited at a wavelength of 375 nm.

On account of structural similarity of CCS and C_10_CN, it is reasonable to deduce that they may form coassembly arrays under certain conditions. In vesicular systems, CCS and C_10_CN were mixed with different molar ratios from 10:0 to 0:10 and a fixed total concentration (10^−4^ m), and the obtained assemblies were characterized by fluorescent emission, circular dichroism (CD), and absorption techniques (**Figure** **5**a; Figure S17a–c, Supporting Information). Surprisingly, it was found that the fluorescent emission intensity for the samples with 10–40 mol% was greatly enhanced (Figure S17, Supporting Information) than those of other ratios. The fluorescent intensity of both CCS and C_10_CN shows increasing tendency upon concentration changes from 10^−6^ to 10^−4^ m. Thus, the fluorescent enhancement of the mixing systems reveals a possibility of coassembly. In coassembly arrays, C_10_CN and CCS molecules form an interdigital arrangement where C_10_CN molecules are isolated, whereby the ACQ effect would be greatly suppressed to give strong emission. In CD studies (Figure 5a), the CCS vesicle system shows a silent Cotton effect at its UV–vis absorption region, but C_10_CN presents a relatively weak positive Cotton effect around its main absorption peak of 455 nm. Unlike C_10_CN, the chirality of cholesterol unit in CCS does not transfer to cyanostilbene group, which could be because of its relatively longer spacer (three carbon atoms) than C_10_CN (two carbon atoms). However, under the appropriate ratios of C_10_CN (30–50 mol%), strong negative Cotton effects appeared at the absorption region of 360–390 nm, which fall in the absorption region of CCS (Figure S17c, Supporting Information). Furthermore, positive Cotton effect at 450 nm assigned to C_10_CN was enhanced accordingly. Thus, it could be proposed that there is considerable synergistic effect between the self‐assemblies.

The chirality during the self‐assembly possesses three levels, namely molecular chirality, chiral amplification (chiral transfer), and macroscopic chirality (or micro/nanoscale chirality that could be recognized by microscope).^43^ Clearly, the formation of spherical vesicles rather than helical/twisted objects demonstrates that the supramolecular chirality only reaches chiral amplification level. The realization of chiral amplification in multicomponent self‐assembly systems follows two principles, either sergeants–soldiers rule or majority rule,^44^ both of which require intimate coassembly between different components. Titration studies by fixing the concentration of one component and increasing the molar ratio of another gradually (Figures 5b; Figures S17d and S18, Supporting Information) were carried out to verify the chirality amplification pathways. The maximum emission wavelength blue‐shifted from 555 to 530 nm upon the addition of CCS to C_10_CN vesicle solution. Absorption peaks broadened and shifted upon the addition of the second component on account of the spectrum overlap. After stepwise addition of C_10_CN into CCS system, adding more than 80 mol% of C_10_CN could be able to induce Cotton effect enhancement, and 40 mol% of CCS in C_10_CN system was capable of arousing strong negative Cotton effect. Sergeants–soldiers rule often occurs when adding a relatively small amount (5–20 mol%) of a chiral additive into an achiral analog. The chiral amplification of the coassembly system normally follows the majority‐like principle (Figure S18d, Supporting Information). Different from the classic majority rule, the presence of strong cooperative effect within the obtained coassemblies induces effective chiral transfer from cholesterol to cyanostilbene moieties.

Next, we examined the self‐assembly behavior of C_10_CN/CCS mixture in gel and crystalline phases. In the gel state, increasing molar ratio of CCS gradually quenches fluorescent emission of C_10_CN organogel (Figure S17e, Supporting Information) without varying its maximum absorption, implying the possible occurrence of intermolecular or interaggregate CT. Though the UV absorbance of CCS was enhanced (Figure S17f, Supporting Information), no variation of the CD spectra could be found even the doping fraction of CCS reached 50 mol% (Figure 5c). Therefore, we speculated that the self‐sorting dominates in the gel phase.

Synergistic coassembly and self‐sorting in vesicle and gel phases were further investigated via morphology studies. Of all mixed samples with differed molar ratios, only spherical vesicles were observed by TEM (Figure 5d–f) and their size distributions are around 100 nm, indicating that the molecular stacking arrays of CCS and C_10_CN form flexible membranous structures. In line with CD studies, CCS and C_10_CN undergo self‐sorting during the aggregation in nonpolar solvents, giving individual crystalline plates and fibrous networks (Figure S17g, Supporting Information). In addition to vesicle and gel phases, the self‐sorting also occurred in the phase of crystalline precipitates (flower structure) induced by moisture according to the CLSM observation (Figure S19, Supporting Information). Based on the macroscopic chirality (M‐type in Figure 3d) and UV/CD spectra of gels, the superhandedness in vesicles would be speculated. At the main UV–vis absorption at 425 nm in the gel phase of C_10_CN (Figure S17f, Supporting Information), the Cotton effect is negative (Figure 5c). Thus, the positive Cotton effect of C_10_CN vesicles (Figure 5a) indicates the P‐handedness. The chiral amplification in co‐assembled vesicle membranes is schematically elucidated in Figure 5g.

### 2.3 Transfer of Light‐Responsiveness and Thermoresponsiveness in Coassembled Vesicles

**Figure 6:**
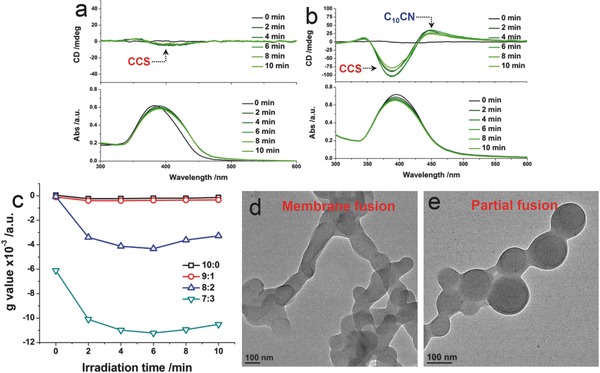
UV light irradiation responsiveness. a,b) CD and UV–vis spectra of samples with molar ratios of 10:0 and 8:2 (CCS:C_10_CN, total concentration: 10^−4^ m) upon increasing the time of UV light irradiation (254 nm). c) Anisotropy factor *g* values of CD spectra at 400 nm calculated by *g* = Δε/ε = θ (mdeg)/(32 980 × A) as a function of irradiation time (interval: 2 min). d,e) TEM images of samples with molar ratios of 10:0 and 8:2 (CCS:C_10_CN, total concentration: 10^−4^ m) under sufficient light irradiation. All samples were prepared in THF–water mixture (*f*_w_ = 90%).

From the experiments in the last section, it was found that C_10_CN and CCS could form intimate coassembly in polar solvent environment. Therefore, it was assumed that the properties of one component might be transferred to another component or to the coassembled vesicles. According to our previous studies,^45^, ^46^ cyanostilbene is photoresponsive (254 nm), whereby its conformation would transform from E‐(*trans*‐) to Z‐(*cis*‐) isomer, leading to the variation of photophysical and self‐assembly behavior. Hereby, we investigated the UV light responsiveness of the build block C_10_CN in the coassembly using absorption and CD techniques. Coassembled vesicle solutions were deposited in quartz cuvettes sealed by parafilm, which underwent periodic UV light irradiation before characterizations. For pure CCS vesicle system, upon increasing UV irradiation time (from 0 to 10 min), absorption spectra displayed slightly reduced absorbance, demonstrating the occurrence of photoisomerization (**Figure** **6**a).^46^ In contrast to the mute Cotton effect before the light irradiation, a weak negative Cotton effect at the absorption area of CCS (around 390 nm) appeared after the irradiation for 2 min, and this CD signal was not enhanced further even under longer irradiation period. Thus, photoisomerization may not effectively enhance the chiral amplification for pure CCS. Next, we introduced C_10_CN with different molar ratios (at a same total concentration of 10^−4^ m) to CCS in order to give coassembled vesicles and examined their UV–vis responsiveness (Figure 6b; Figure S20a–c, Supporting Information). For the samples with C_10_CN ratios of 10 and 20 mol%, there were no active CD signals before the UV light treatment, and active CD signals appeared after the UV light treatment. Significantly, the coassembly with 20 mol% C_10_CN (Figure 6b) showed strong Cotton effect (up to 100 mdeg from 0). In addition, we observed the CD signal enhancement at the C_10_CN absorption area (450 nm), meaning that light‐responsiveness was transferred to the C_10_CN moiety. When the molar fraction of C_10_CN reached 30 mol%, the pristine system possessed relatively strong CD signals, which could be enhanced by UV light irradiation as well. After adding more than 50 mol% C_10_CN, the coassemblies were insensitive to the UV light irradiation even after long period (Figure S20c, Supporting Information). The anisotropy factor *g* value at 400 nm was summarized as a function of irradiation time (Figure 6c), which clearly illustrates the UV light‐triggered chiral amplification of CCS.

To the best of our knowledge, this is the first coassembly system of which superchirality could be greatly enhanced by photoirradiation, and the light‐responsiveness could be effectively transferred to light‐insensitive species. Several factors contribute to this particular phenomenon. The synergistic coassembly and the formation of mixed molecular arrangement array are keys to the photoresponsive chirality manipulation, where chiral dopant (C_10_CN) and photosensitive moiety (CCS) are required. Photoisomerization results in the conformational variation of cyanostilbene as well as the perturbation and destructive influence to the molecular order. It is reminiscent of chiral azobenzene‐doped liquid crystal, of which chirality can be controlled by photo irradiation.^47^, ^48^ Similarly, the stilbene moiety shares most of the characteristics with that of the azobenzene unit. In contrast to relatively flat conformation of the E‐isomer, the Z‐isomer of cynaostilbene possesses statically twisted aromatic rings, which can significantly improve the chirality transfer. In addition, bent shaped E‐isomer has a greatly reduced dihedral angle of 8.9° as compared to linear shaped *Z*‐isomer of 177.91°,^46^ suggesting that free rotation of stilbene is restricted after the photoisomerization. Consequently, the Z‐isomer owns considerable molecular rigidity and steric hindrance, facilitating the superchirality transfer. Apart from the influence to the chirality amplification, photoisomerization led to morphological variations as well (Figure 6d,e; FigureS20d,e, Supporting Information). Pure CCS vesicles undergo the membrane fusion to form branched nanochannels upon the photoisomerization, the mechanism of which has been elucidated in our last report utilizing another cyanostilbene derivative.^33^ After being mixed by C_10_CN (20 and 50 mol%), however, only partially fused/adhered vesicles were obtained. When C_10_CN was dominant in the coassembly, vesicle fusion/adhesion totally disappeared, which was replaced by the shape deformation (Figure S20, Supporting Information). At high molar ratio of C_10_CN (80 mol%), CCS molecule was isolated as monomeric state in the C_10_CN stacking array, which fails in inducing the membrane fusion process. The membrane deformation might be accounted for the perturbation stemmed from conformational variation of cyanostilbene.

Since C_10_CN self‐assembly is thermally responsive, its thermal properties were investigated spectroscopically via absorption/CD techniques. Nanofiber formation and gelation in nonpolar solvents exhibited supramolecular polymerization behavior as stated above. Thus, a dilute decane solution of C_10_CN was subjected to a cooling cycle (cooling rate: 0.5 K min^−1^) when the absorption and CD signals were monitored at 2 °C intervals (Figure S21a, Supporting Information). With decreasing temperature, the absorption peak at 417 nm decreased gradually and red‐shifted to 428 nm with a shoulder peak at 446 nm, indicating the temperature‐responsive aggregation. For CD spectra, it showed silent Cotton effect at relatively high temperatures, while a negative Cotton signal at 427 nm emerged at a temperature of 291 K, indicative of the formation of nanofibers. Further decreasing the temperature enabled red shift of CD peaks from 450 to 485 nm, as a result of electrostatic interaction between stacked chromophores in a shoulder‐to‐shoulder arrangement. The degree of aggregation (α_agg_ = (CD − CD_mon_)/(CD_agg_ − CD_mon_)) was calculated, which was then plotted against temperature (Figure S21b, Supporting Information). Such a sigmoidal curve, fitted well with Boltzmann equation (*R* ^2^ = 0.99), is the characteristic of isodesmic chain growth model.^49^ The isodesmic self‐assembly pathway of C_10_CN in nonpolar solvents was also supported by absorption‐based α_agg_‐temperature profile (Figure S21c, Supporting Information). Some thermodynamic parameters could then be deduced from this model. For example, the enthalpy release during the chain elongation is Δ*H* = −969.2 kJ mol^−1^ with an equilibrium constant (*K*_e_ = 1.25 × 10^3^ m) at 290 K.

**Figure 7:**
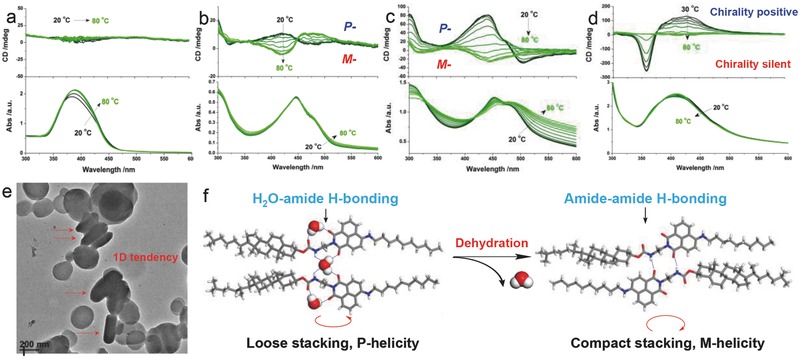
Thermoresponsiveness. a–d) CD and UV–vis spectra of CCS (10^−4^ m), C_10_CN (5 × 10^−5^ m), C_10_CN (10^−4^ m), and CCS–C_10_CN mixture (total concentration: 10^−4^ m, molar ratio: 5:5) with 10 °C interval. e) TEM images of C_10_CN assemblies at a high temperature (90 °C), where red arrows indicate the emergence of rod‐like aggregates. All samples were prepared in THF–water mixture (*f*_w_ = 90%). f) Effect of heating‐induced dehydration on supramolecular helicity inversion: a proposed mechanism.

After that, the thermoresponsive properties of CCS and C_10_CN assemblies in polar solvents were evaluated. In a well‐sealed cuvette, vesicular solution was gradually heated up from room temperature to 80 °C. Though absorption spectra of CCS vesicle system displayed redshift and the increase in absorbance contributed by the partial disorganization at high temperature, no active Cotton effect signals could be observed (**Figure** **7**a), indicative of thermoinsensitiveness of CCS in polar solvents. In direct contrast, when being heated, C_10_CN vesicle solution was accompanied with helicity inversion (Figure 7b,c). In a relatively dilute state (5 × 10^−5^ m), CD spectra of C_10_CN showed a positive Cotton effect peak at 420 nm, corresponding to the absorption of naphthalimide chromophore, which is assigned as P‐helicity according to our above analysis. Nevertheless, CD signals converted from positive to negative gradually to generate complete mirror‐spectrum, suggesting the inversion of handedness in C_10_CN assemblies (M‐helicity). Only a minor red shift occurred in its absorption spectra upon heating. In order to gain insight into the chirality inversion, the concentration of C_10_CN was elevated to 10^−4^ m. This concentration also presented the helicity inversion with a tiny red shift from 440 to 446 nm (Figure 7c). The red shift of CD spectra is due to the red shift of corresponding absorption spectra. As displayed in the bottom image of Figure 8c, the absorbance at 450 nm decreased, while the peak at 490 nm increased accordingly (Figure S22, Supporting Information). The great peak shift (≈40 nm) strongly indicates molecular rearrangement to giving rise to more closely π‐stacked arrays, which is induced by heating. Afterward, further characterizations revealed successful transfer of thermoresponsiveness from C_10_CN to CCS in mixed vesicle membranes (Figure 7d). In coassembled vesicles, the CD signal assigned to C_10_CN at 428 nm and the CD signal belonging to CCS at 360 nm disappeared stepwise without the observation of inversion. We believed that the hydration/dehydration of vesicular inner core is important to the helicity inversion and helicity silence upon heating.

Based on our moisture‐sensitivity studies, C_10_CN could easily capture a water molecule via hydrogen bonding even in the presence of ultralow moisture content. Thus, in polar aqueous media, C_10_CN would surely bond with water that occupies at least two hydrogen‐bonding sites to result in loosely packed arrangement. In anhydrous nonpolar solvent (gel phase), the mutual impact of strong interamide (imide) hydrogen bonding (Figure S16, Supporting Information) and chiral cholesterol unit generates a preferred orientation of M‐helicity. On the other hand, after C_10_CN coordinates with water, interamide (imide) hydrogen bonding is replaced by H_2_O–amide/imide hydrogen bonding with a varied orientation, resulting in the occurrence of P‐helicity. Under the high temperature condition, water molecule would escape from vesicle membranous inner core, facilitating the reformation of interamide (imide) hydrogen bonding to give the handedness identical to gel phase (M‐helicity). Interamide (imide) hydrogen bonding favors the formation of 1D aggregates, and indeed we observed the emergence of 1D rod‐like vesicles/nanoparticles (red arrows in Figure 7e). The dehydration‐induced helicity inversion was observed in Figure 7f. In some reported helical inversion systems, the participation of water has a vital importance. For instance, Fenniri et al. elucidated the role of water in chiral inversion by utilizing the chiral guanine and cytosine self‐assembly system.^50^ They found that water could help establish a hydrogen bonding network and change the conformation of building blocks in the self‐assembly geometry. Lee and coworkers^51^ revealed the temperature‐induced dehydration effect in helical inversion of self‐assembled nanotubes, and similar to our study, they believed that the enhanced π–π stacking after the dehydration was the main reason for the helicity inversion. In a coassembled system, temperature‐variable absorption spectra only displayed a slight blue shift, indicative of the absence of enhanced π–π stacking. The interdigitation between CCS and C_10_CN hinders the formation of interamide hydrogen bonding, and thus, upon the dehydration, only mute CD signals were shown in CD spectra.

## 3 Conclusions

In summary, a two‐component supramolecular system based on C_10_CN and CCS has been constructed, and their individual self‐assemblies as well as coassemblies have been systematically studied. The self‐assemblies exhibit diverse topological morphologies according to different solvent environments. Self‐sorting and coassembly scenarios occur in nonpolar solvents, including the formations of gel and crystalline phases. While in the vesicle phase in aqueous media, C_10_CN and CCS give mixed self‐assembly arrays, where they share stimulus responsiveness. As a result of the unique coassembly, the properties of photoinsensitive C_10_CN and thermoinsensitive CCS exchange with each other, exhibiting photoresponsiveness and thermoresponsiveness, respectively. The present work sheds light on how to transfer intrinsic properties between components within coassembled systems.

## 4 Experimental Section

Experimental details including reagent information, instrument characterization of self‐assemblies, and preparation of samples can be found in the Supporting Information.

[CCDC 1533821 (C_10_CN) and 1533820 (CCS), contain the supplementary crystallographic data for this paper. These data can be obtained free of charge from The Cambridge Crystallographic Data Centre via www.ccdc.cam.ac.uk/data_request/cif.].

## Conflict of Interest

The authors declare no conflict of interest.

## Supporting information

Supplementary
