# Supplementary material for: Environment‐Adaptive Coassembly/Self‐Sorting and Stimulus‐Responsiveness Transfer Based on Cholesterol Building Blocks
Source: Adv Sci (Weinh). 2017 Nov 8;5(1):1700552. doi: 10.1002/advs.201700552 (PMC5770671; doi:10.1002/advs.201700552)
Supplement: Supplementary file 1 — Supplementary [file ADVS-5-na-s001.pdf]

Copyright WILEY-VCH Verlag GmbH & Co. KGaA, 69469 Weinheim, Germany, 2017.

Supporting Information

for *Adv. Sci.,* DOI: 10.1002/advs.201700552

Environment-Adaptive Coassembly/Self-Sorting and Stimulus-Responsiveness Transfer Based on Cholesterol Building Blocks

*Pengyao Xing, Huijun Phoebe Tham, Peizhou Li, Hongzhong Chen, Huijing Xiang, and Yanli Zhao**

Supporting Information

Materials and methods

All starting chemicals and solvents were purchased from Aladdin Medicine Reagent Co. Ltd. and used without any further purifications. Cyanostilbene and $C_{10}CN$ precursor CN were synthesized according to our previous reports.[S1,S2] Crystal CIF files for CCDC 1533821 ($C_{10}CN$) and 1533820 (CCS).

Synthesis of $C_{10}CN$. CN (360 mg, 0.5 mmol) and decylamine (780 mg, 5 mmol) were added into DMSO (20 mL). The mixture was heated at 100 ^{o}C with stirring under N_2 protection for 8h. After cooling down to room temperature, the mixture was poured into ice water, which was then extracted by ethyl acetate three times. The extracted solution was dried by $MgSO_4$, and concentrated sample was subjected to column chromatography (eluent: from 1000/1 to 100/1, DCM/MeOH) to afford pure $C_{10}CN$ (~300 mg, yellow powder, yield 75%). ^{1}H NMR (300 MHz, $CDCl_3$, 298 K). δ = 8.57 (d, 1H), 8.48 (d, 1H), 8.06 (d, 1H), 7.62 (t, 1H), 6.74 (d, 1H), 5.23 (d, 3H), 4.37 (t, 3H), 3.55 (t, 2H), 3.41 (m, 2H), 2.3-0.66 (m, 62H). ^{13}C NMR (100 MHz, $CDCl_3$, 298 K) δ = 165.09, 164.50, 156.39, 149.81, 139.91, 134.75, 131.23, 129.88, 125.12, 124.46, 122.64, 122.20, 120.07, 109.49, 104.24, 74.14, 56.66, 56.12, 49.96, 43.77, 42.29, 40.68, 39.72, 39.51, 39.35, 38.42, 36.95, 36.48, 36.18, 35.79, 31.89, 29.60, 29.32, 28.01, 24.27, 23.83, 22.82, 22.68, 22.56, 21.00, 19.31, 18.7, 12.12, 11.83. HRMS (TOF) m/z $[M+H]^+$, calcd for $C_{52}H_{78}N_3O_4$, 808.5969; found, 808.5992.

Synthesis of CCS. Cholesteryl chloroformate (450 mg, 10 mmol) was dissolved in DCM (20 mL) at 0 ^{o}C, which was dropped into a DCM (30 mL) solution of 3-bromopropylamine hydrochloride (218 mg, 10 mmol) with triethylamine (100 μL). After the reaction for ca. 30 min, the mixture was filtered. The transparent DCM solution was subjected to rotary evaporator to remove solvents, and the resultant solid was used for the next reaction directly. This compound was mixed with cyanostilbene (130 mg, 5 mmol) and K_2CO_3 (100 mg) in acetone (50 mL), and the mixture was refluxed overnight. The resultant solution was then filtered and subjected to rotary evaporation to remove all solvents. The crude product was re-crystalized in DCM/hexane for three times to afford the final product (200 mg, yellow

powder, yield: 54%). ^{1}H NMR (300 MHz, $CDCl_3$, 298 K). δ = 7.80 (d, 2H), 7.53 (d, 2H), 7.26 (s, 1H), 6.90 (d, 2H), 6.72 (d, 2H), 5.37 (s, 1H), 4.82 (s, 1H), 4.48 (m, 1H), 4.10 (t, 2H), 3.40 (d, 2H), 3.05 (s, 6H), 2.3-0.66 (m, 44H). ^{13}C NMR (100 MHz, $CDCl_3$, 298 K) δ = 158.73, 151.57, 148.72, 140.85, 136.83, 131.62, 129.58, 128.35, 127.57, 126.76, 125.46, 122.49, 121.88, 119.52, 117.21, 114.84, 113.10, 111.66, 111.27, 109.59, 104.42, 74.37, 70.99, 65.92, 59.92, 56.69, 50.52, 45.38, 42.32, 40.07, 39.52, 38.35, 37.00, 36.19 34.59, 31.88, 30.34, 29.50, 28.02, 27.39, 24.29, 23.84, 22.97, 21.05, 19.33, 18.72, 14.21, 11.86, 7.74, . HRMS (TOF) m/z $[M+H]^+$, calcd for $C_{52}H_{78}N_3O_4$, 808.5969; found, 808.5992.

Preparation methods of assemblies. Stock solutions of C_{10}CN and CCS in organic solution (THF) with the concentration of 10 mM were first prepared. A small portion of stock solution was injected into deionized water, decane or hexane followed by the sonication and aging to obtain different self-assembly systems. Notably, all solutions were aged at least 24 h before characterizations.

Characterizations

^{1}H NMR spectra were measured on a Bruker-AC300 spectrometer. ^{13}C NMR spectra were measured on a Bruker BBFO-400 spectrometer. High-resolution mass spectrometry (HR-MS) was performed on a Waters Q-tof Premier MS spectrometer. Transmission electron microscopy (TEM) was recorded through a JEM-1400 (JEOL, 100kV). In the TEM sample preparation, ca. 30 μL self-assembled solution was dropped on TEM copper grids, followed by drying in air. HRTEM studies were carried out on a JEM-2010 (JEOL, 200 kV). Scanning electron microscopy (SEM) samples were tested by a field-emission JSM-6700F (JEOL). Samples for SEM tests were prepared by dropping about 100 μL self-assembled solution on the polished silicon wafer, followed by sucking most of liquids and air drying. Powder X-ray diffraction patterns were obtained via a Bruker D8 powder X-ray diffractometer at 40 kV and 30 mA using Cu Kα radiation (λ = 1.5418 Å). Samples for Fourier transform infrared (FT-IR) measurements were recorded on an Avatar 370 FT-IR spectrometer. Circular dichroism (CD) spectra were measured with a Jasco J-810 CD spectrophotometer. For the rheological measurements, the gels were tested by a Thermo Scientific HAAKE Rheo-Stress 6000. The emission spectra were obtained *via* a Shimadzu RF-5301pc fluorescence spectrophotometer, and UV-vis absorption spectra were recorded using a Shimadzu UV-3600 spectrophotometer.

Single crystals with appropriate sizes were chosen under an optical microscope, coated with vacuum grease to prevent deconstruction, and mounted on a glass fiber for data collection on a SuperNova X-ray diffraction system from Agilent Technologies at 293 K. Simulated molecular arrangements were obtained *via* the software Materials Studio 7.0 by Accelrys.

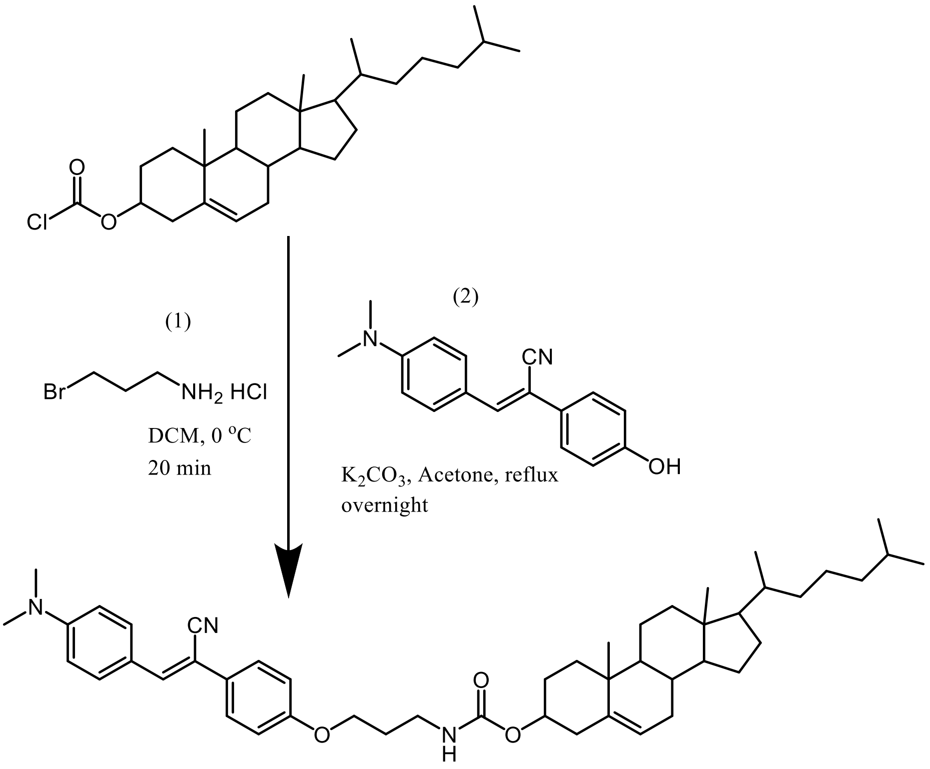

Scheme S1 Synthetic route of CCS.

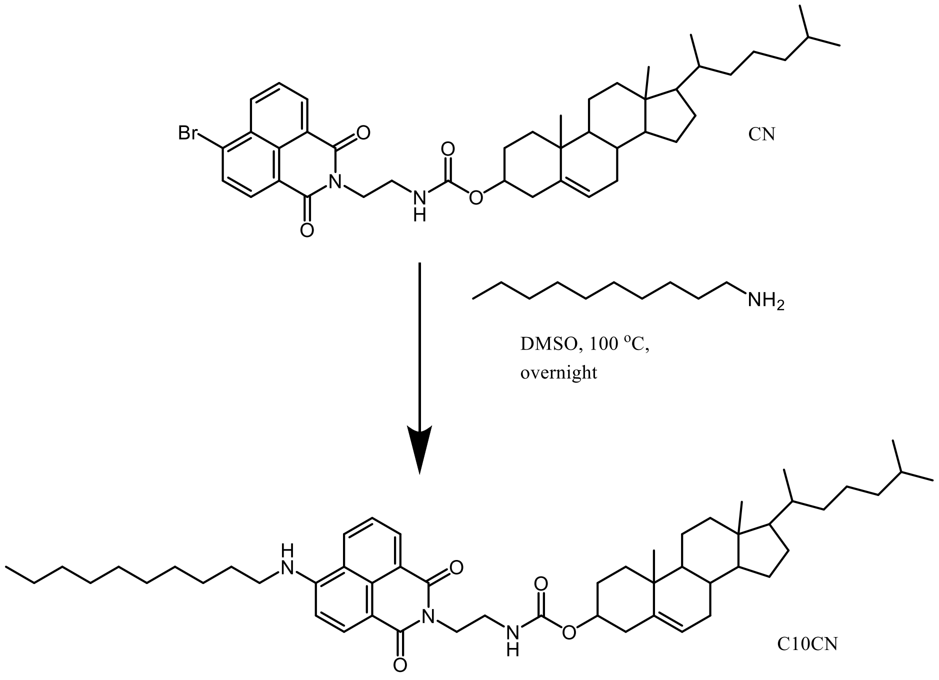

Scheme S2 Synthetic route of C_{10}CN.

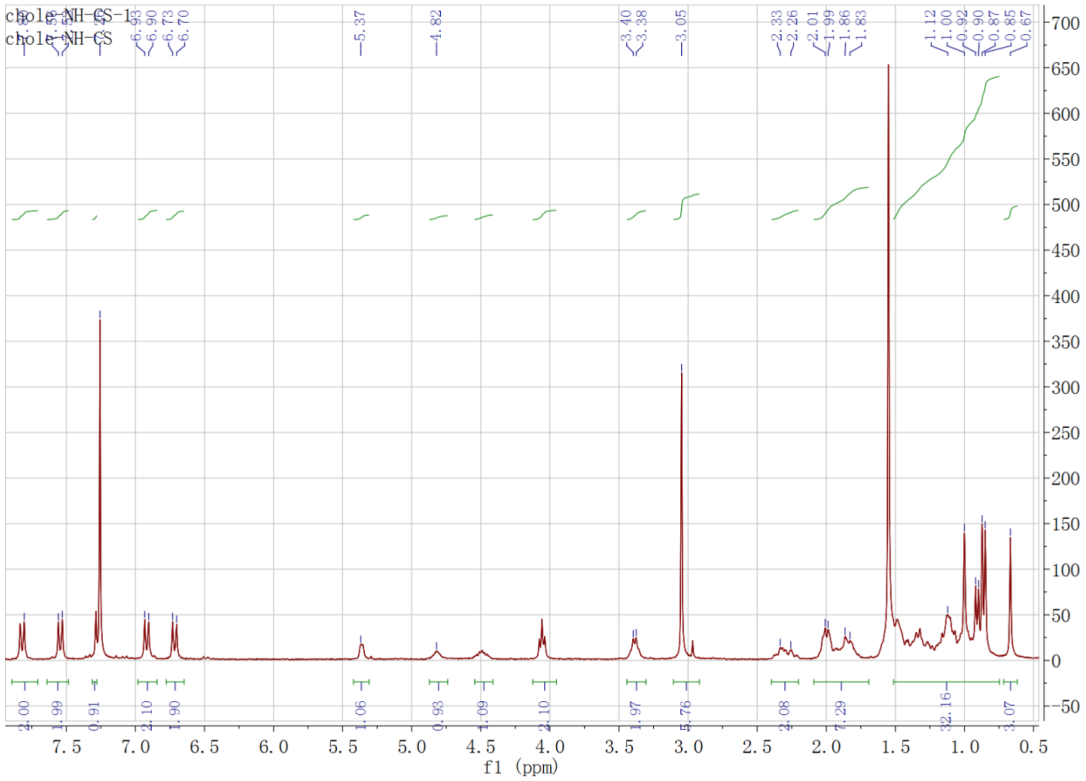

Figure S1 ^{1}H NMR spectrum of CCS.

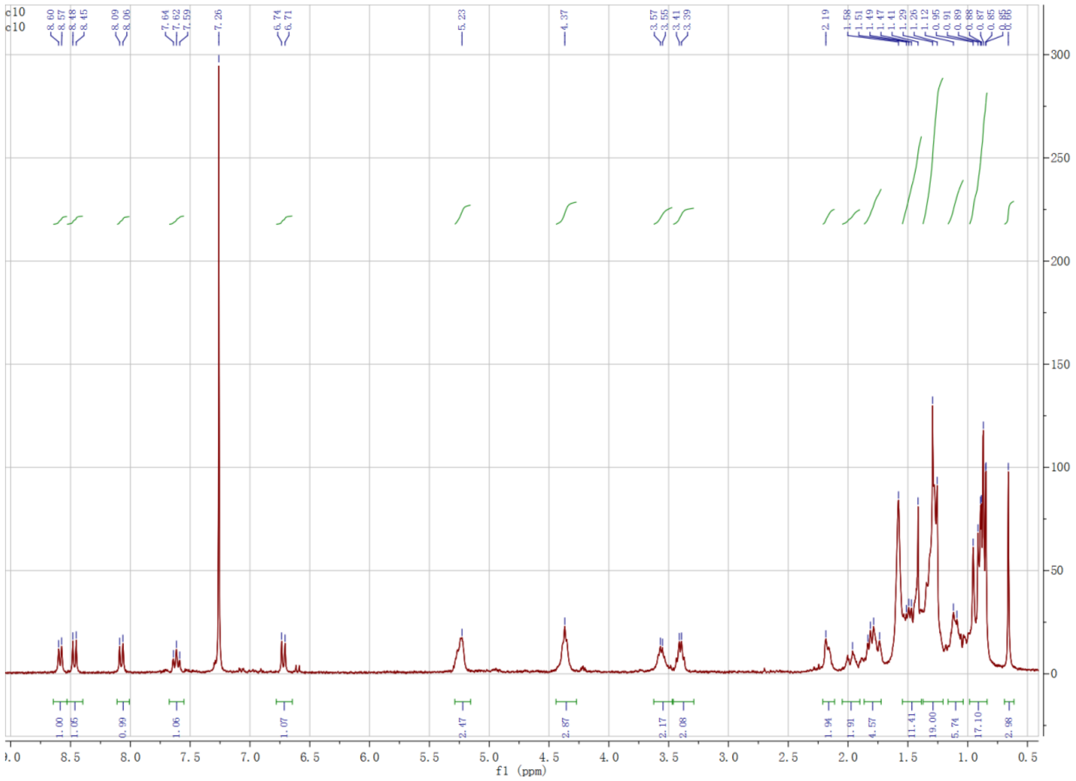

Figure S2 ^{1}H NMR spectrum of $C_{10}CN$.

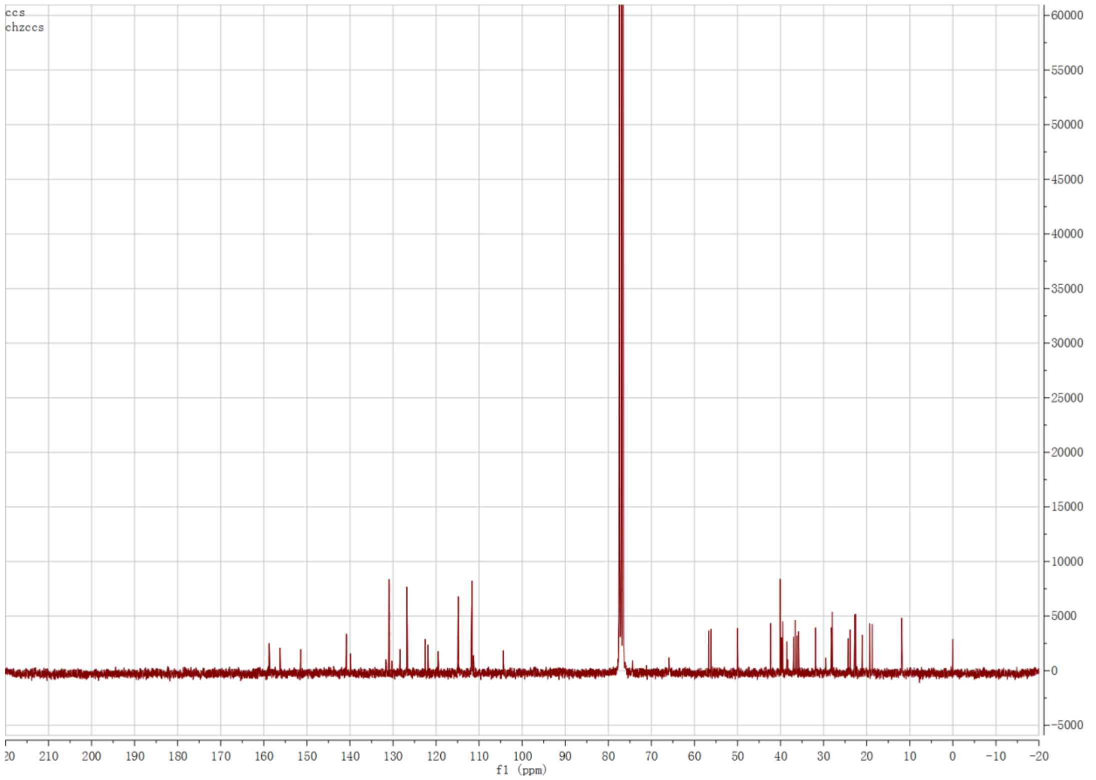

Figure S3 ^{13}C NMR spectrum of CCS.

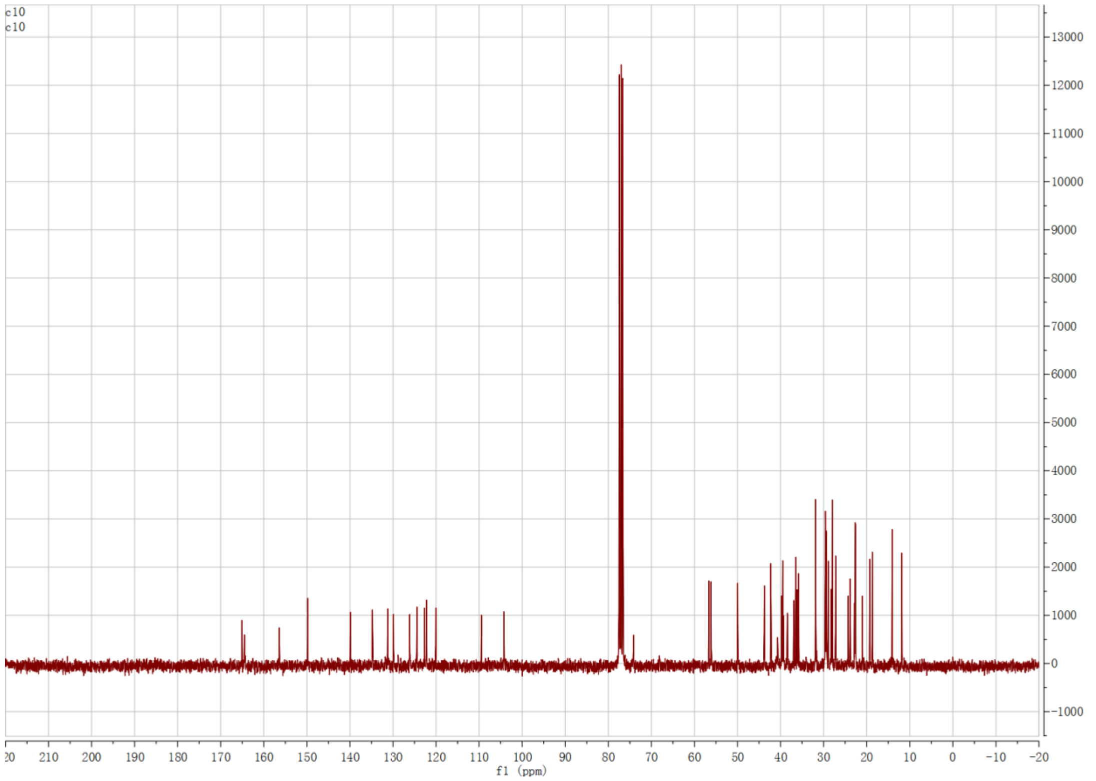

Figure S4 ^{13}C NMR spectrum of $C_{10}CN$.

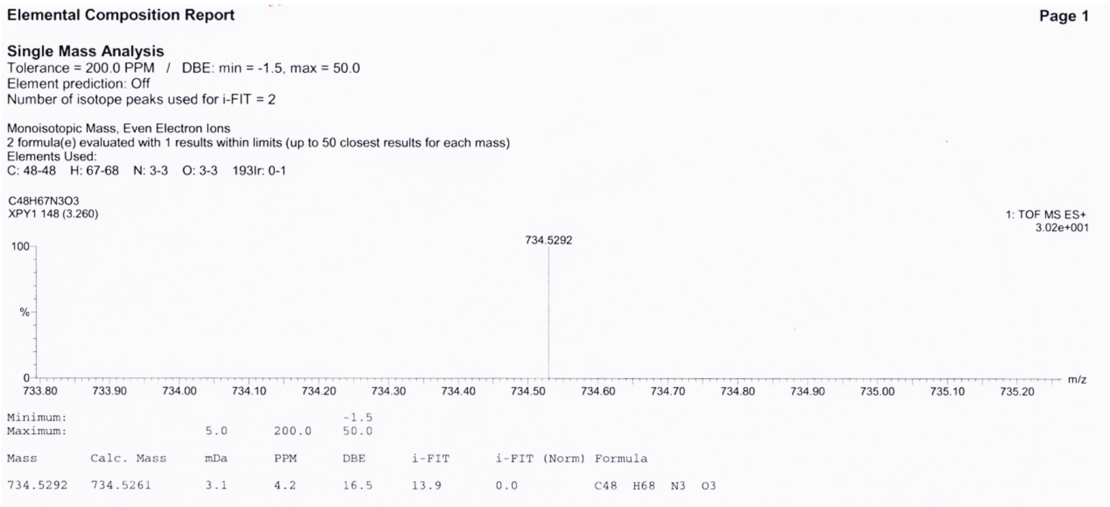

Figure S5 HRMS spectrum of CCS.

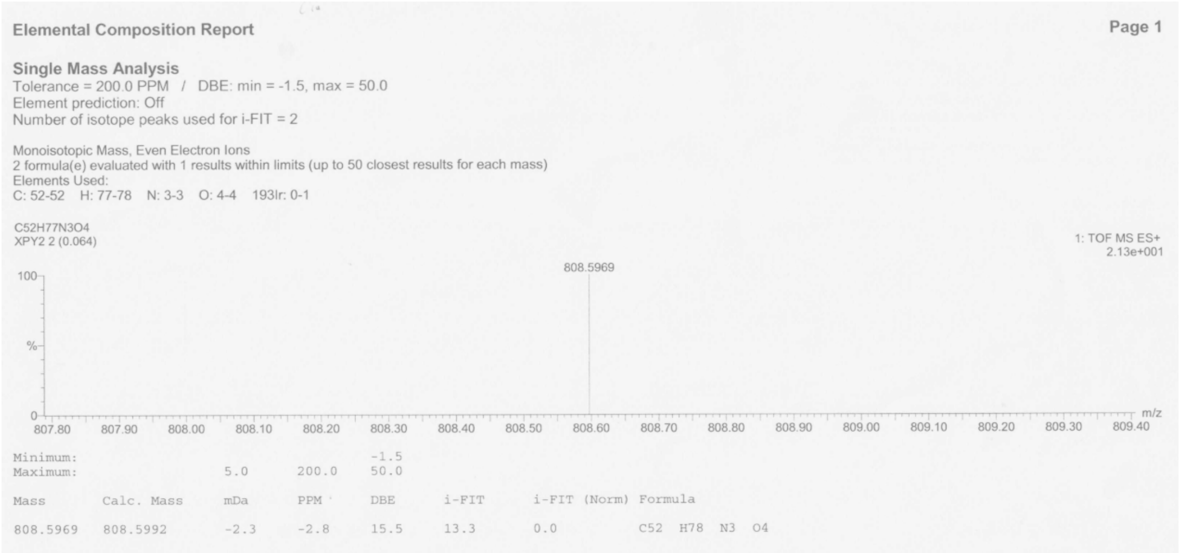

Figure S6 HRMS spectrum of $C_{10}CN$.

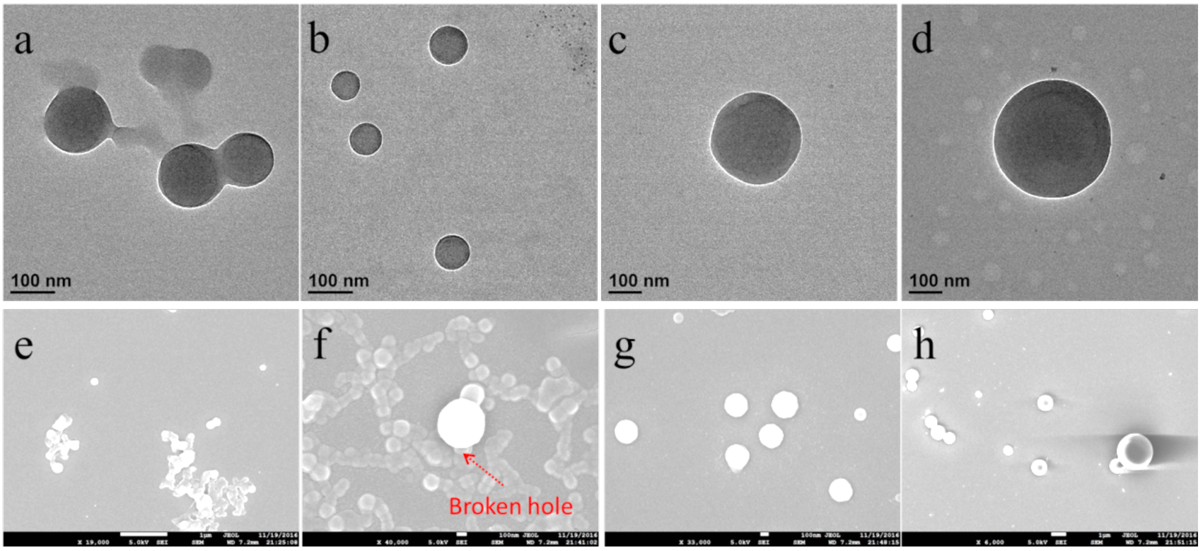

Figure S7 (a-d) TEM images of CCS vesicles with different water fractions (90 vol%, 80 vol%, 70 vol%, and 60 vol%, resepectively). (e-h) Corresponding SEM images.

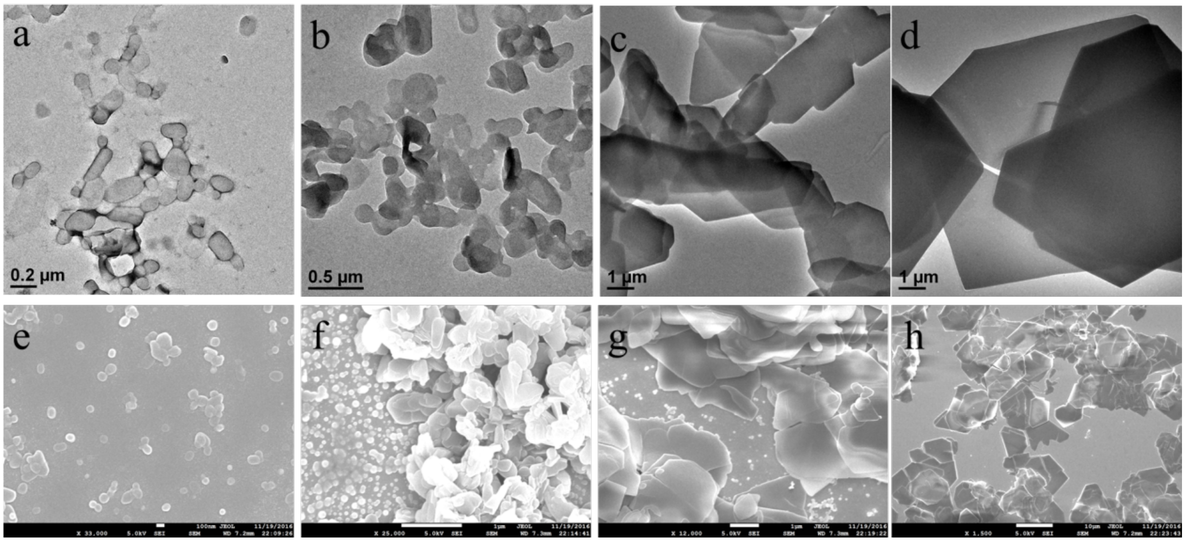

Figure S8 (a-d) TEM images of $C_{10}CN$ vesicles and micro/nano-plates with different water fractions (90 vol%, 80 vol%, 70 vol%, and 60 vol%, respectively). (e-h) Corresponding SEM images.

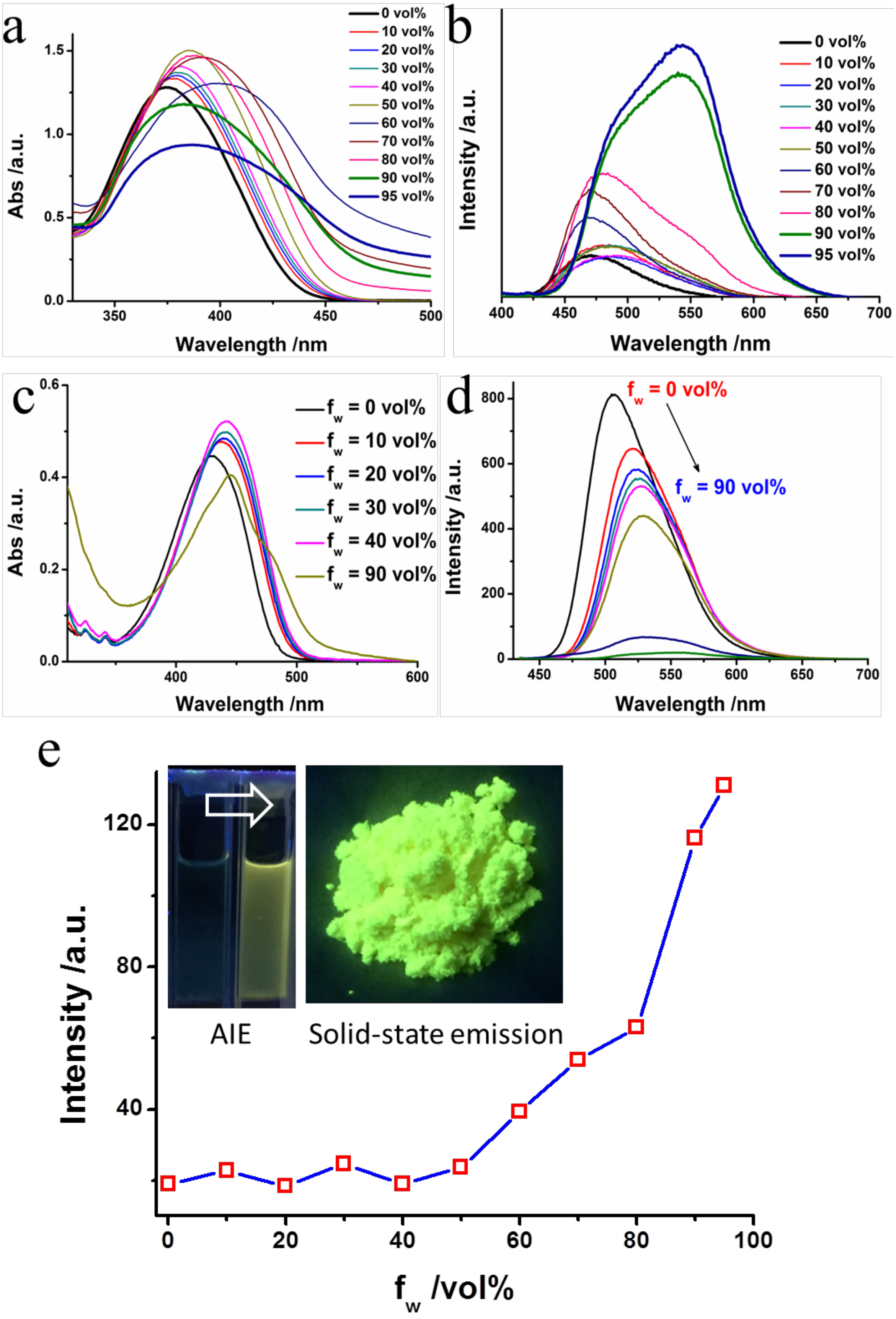

Figure S9 Water fraction-dependent UV-vis absorption and fluorescent emission spectra of

(a,b) CCS and (c,d) $C_{10}CN$, the concentration of which was fixed at 10^{-4} M. (e) Fluorescent emission intensity variation of CCS (10^{-4} M) with the increase of water fractions. Insets show the aggregation-induced-emission (AIE) effect after the self-assembly as well as the solid-state bright emission. The self-assembly in polar solvents had profound effects on the spectroscopic spectra. As compared to the monomer state in THF, the increase in f_w resulted in broadening, red-shifted, and decreased absorbance in absorption spectra (Figure S9a,c). Therefore, *J*-type π-π stacking was formed during the self-assembly. Supramolecular aggregation enabled red-shifted emission spectra of CCS from 470 nm to 550 nm with enhanced intensity (Figure S9b). The emission intensity was elevated by a factor of 6, exhibiting the AIE effect that was also revealed by bright solid-state emission (Figure S9e). In contrast, the fluorescence emission intensity of $C_{10}CN$ was greatly quenched when water was added, showing classic aggregation-caused-quenching (ACQ) effect (Figure 9d).

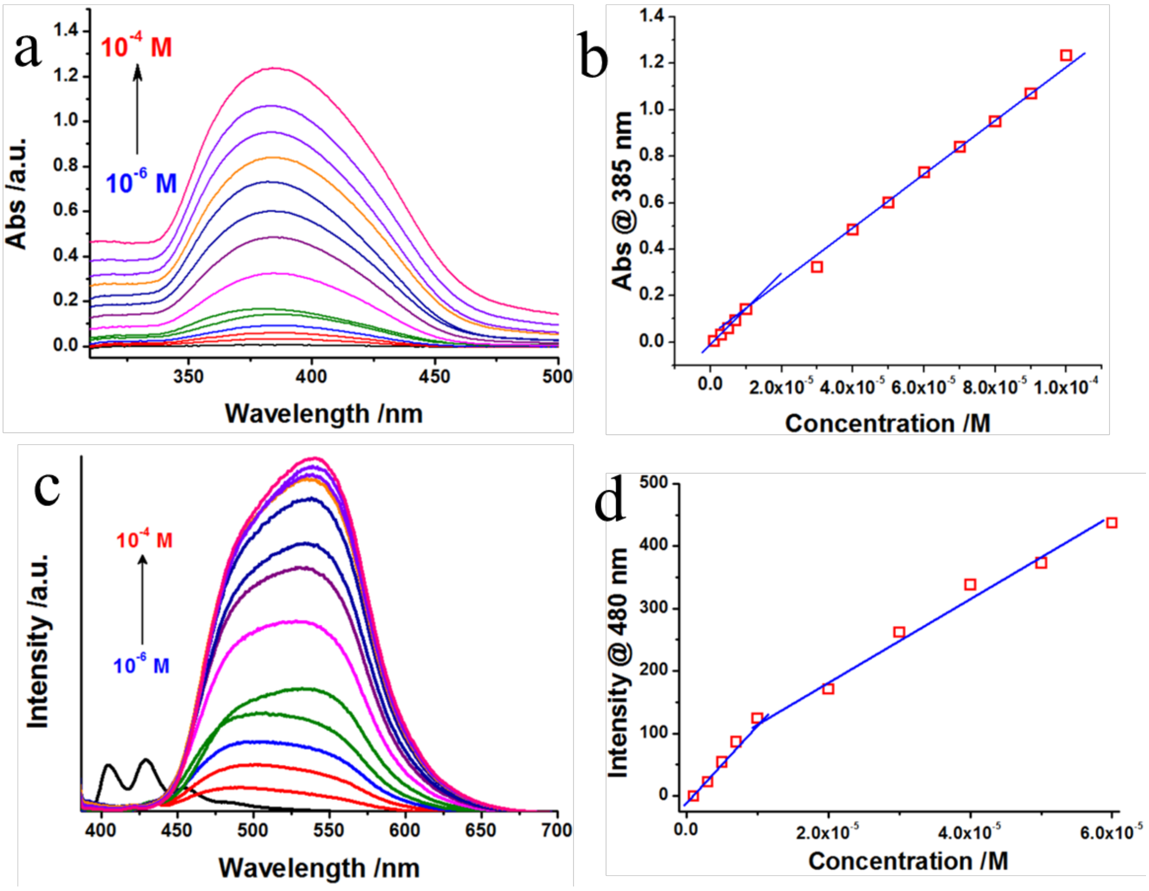

Figure S10 Concentration-dependent absorption (a,b) and emission (c,d) spectra of CCS in THF-water mixture (f_w = 90 vol%).

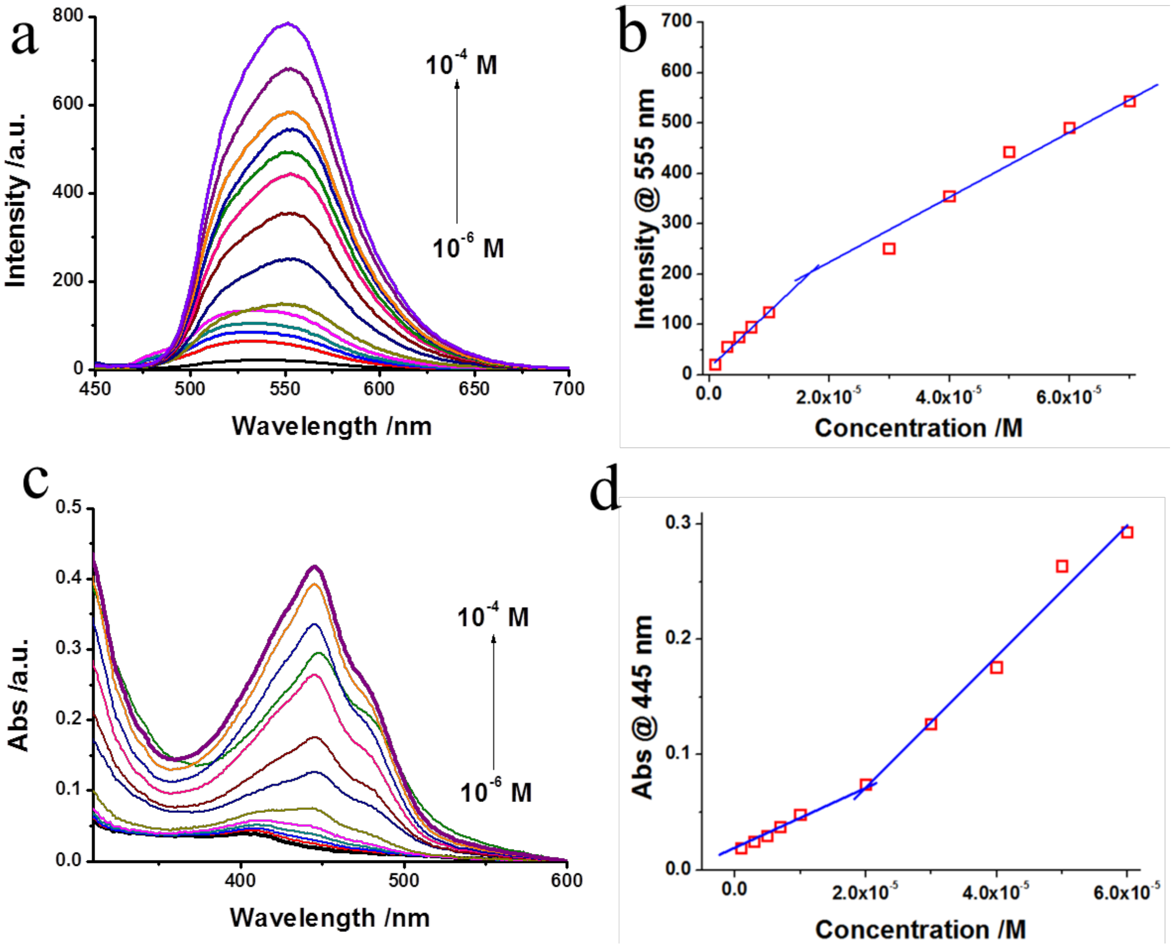

Figure S11 Concentration-dependent emission (a,b) and absorption (c,d) spectra of $C_{10}CN$ in THF-water mixture (f_w = 90 vol%).

Discussions regarding CAC

At a quite low concentration, most building blocks stay in monomeric state. They transform into the aggregation state above a certain concentration namely critical aggregation concentration (CAC). The absorbance at 385 nm and emission at 480 nm were chosen as monomeric peaks of CCS, both of which slopes exhibited mutant points at ca. 10^{-5} M (CAC). Similarly, the absorbance at 445 nm (aggregation) and emission intensity at 555 nm (monomer) for $C_{10}CN$ as the function of concentration displayed mutant points at around 2×10^{-5} M (CAC).

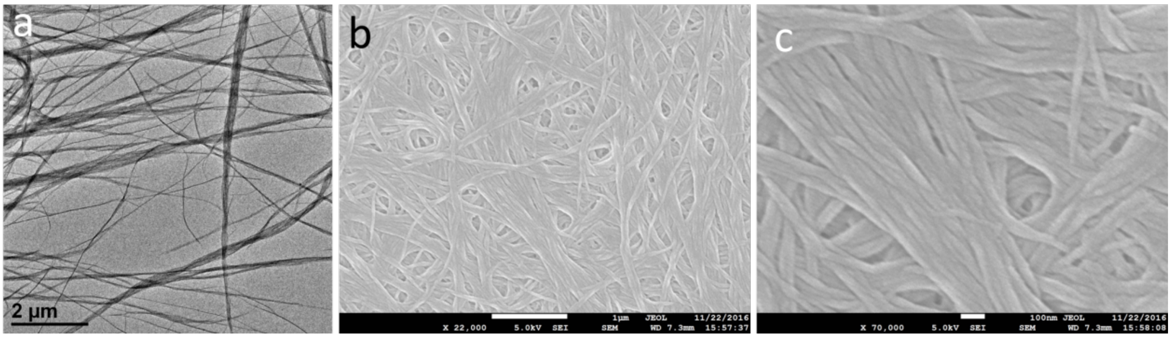

Figure S12 (a) TEM and (b,c) SEM proofs of "bundle" effect in $C_{10}CN$ gels.

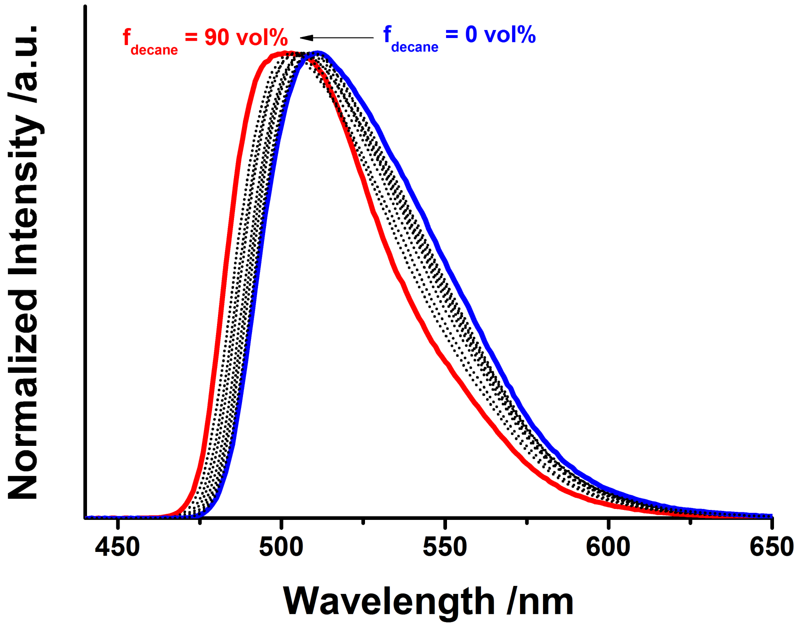

Figure S13 Normalized emission spectra of $C_{10}CN$ with the increase of decane fraction in THF.

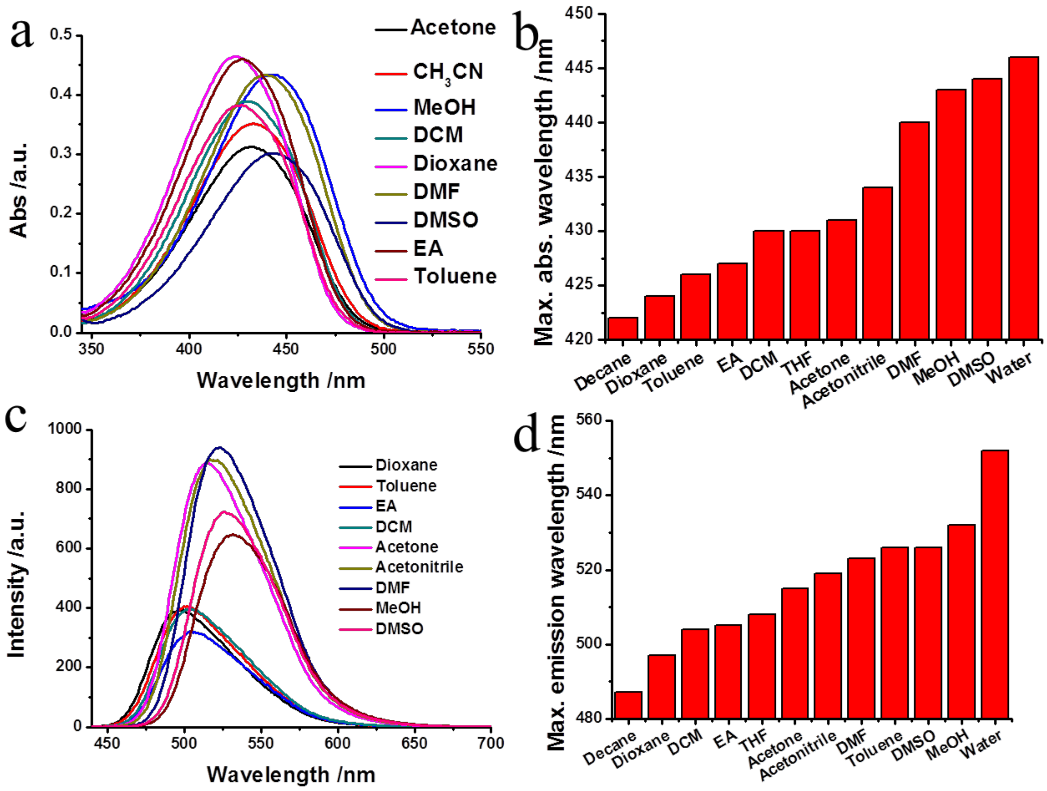

Figure S14 Emission and absorption spectra of $C_{10}CN$ in different solvents with the same concentration (10^{-4} M).

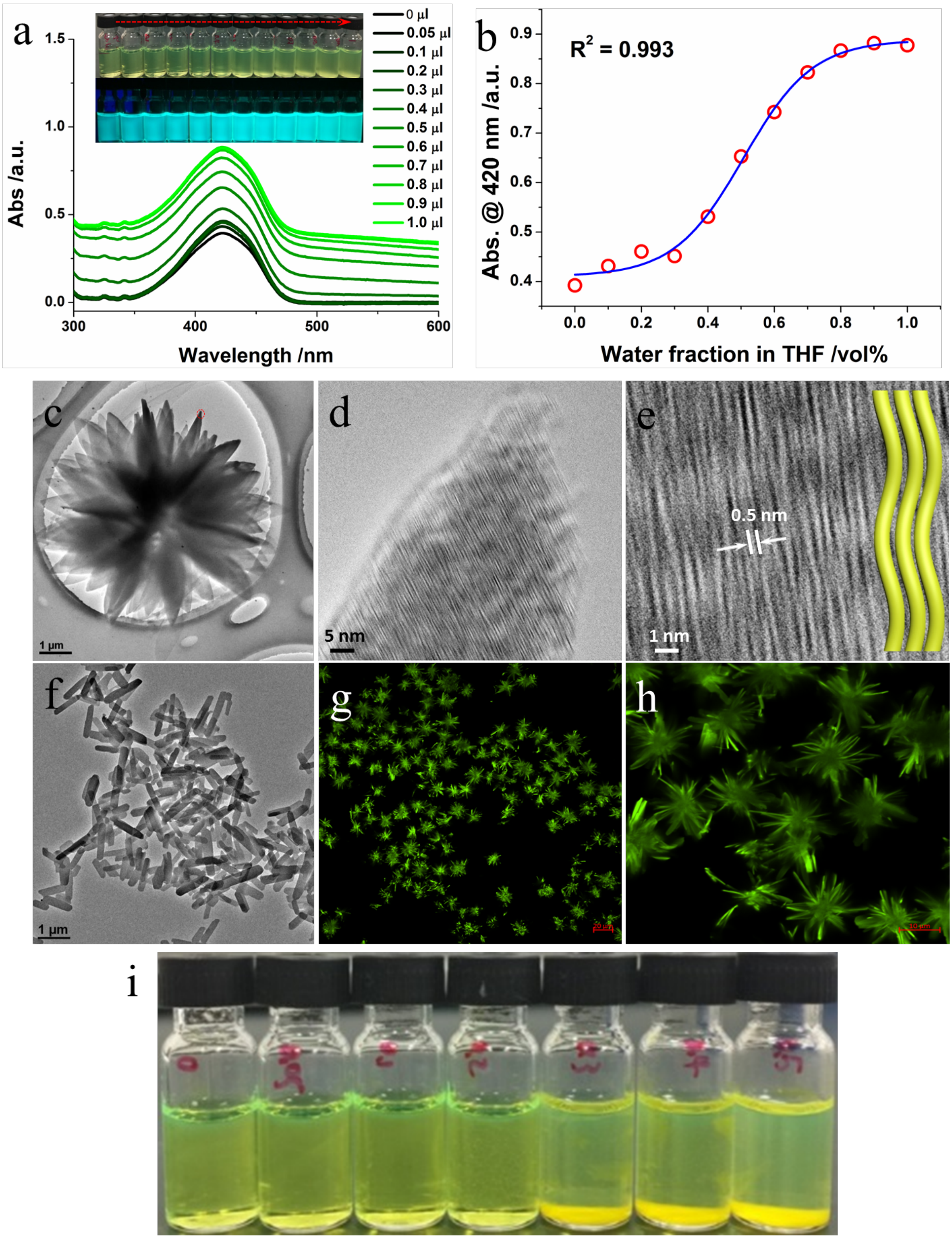

Figure S15 (a) Effects by trace amount of water. Water content-dependent UV-vis absorption spectra of CCS in THF-decane mixture (1:9 vol/vol, 10^{-4} M). (b) Absorbance at 420 nm as a function of water percentage in THF. (c-e) TEM and HRTEM images with different magnifications for microflowers induced by water (0.2 vol% water, concentration of $C_{10}CN$: 1

mM). Inset of (e) stands for the schematic model of helical units observed from the HRTEM images. (f) TEM image of nanoplates (0.1 vol% water, concentration of $C_{10}CN$: 2 mM). (g,h) CLSM images of microflowers (0.1 vol% water, concentration of $C_{10}CN$: 0.5 mM). (i) Digital images of $C_{10}CN$ self-assembled system in decane (with 10 vol% THF) with the increase of water content. From left to right: 0 vol%, 0.005 vol%, 0.01 vol%, 0.02 vol%, 0.03 vol%, 0.04 vol%, and 0.05 vol%.

Discussions regarding moisture sensitivity

We utilized fresh prepared anhydrous THF (10 vol%) with different amounts of pre-added water to study the moisture influence on the phase behavior. From the digital images of $C_{10}CN$ self-assembled systems in decane (with 10 vol% THF) upon increasing the water content (Figure S15i), it could be observed that the gel phase started to collapse when 0.02 vol% water was added, and precipitates were obtained after adding more than 0.03 vol% water. The observations indicate that the gelation is ultra-sensitive to moisture. We then tried to evaluate if the $C_{10}CN$ self-assembly system could serve as a moisture sensor. A low concentration (10^{-4} M) of $C_{10}CN$ without rapid precipitation or gelation was employed (Figure S15a). With the increase in the moisture amount from 0 to 1 μL (1 mL of total volume), the turbidity became more and more obvious (inset of Figure S15a), yet the fluorescent emission variation was irregular. By summarizing the absorbance value as a function of water volume fraction (Figure S15b), the obtained curve fits Boltzmann model with an R^2 value of 0.993. Therefore, the system could serve as a supramolecular platform for moisture-sensing in water miscible solvents, and the limit of detection (LOD) that is defined as 10 % of absorbance change is determined to be 0.26 vol%. Next, we characterized morphologies of moisture-induced precipitates (Figures S15c-h and S16). At the low concentration range of $C_{10}CN$ (such as 0.5 mM or 1 mM), a trace amount of water (0.1 vol%) induces the formation of flower-like microstructures, confirmed by TEM and CLSM. The relatively sharp edges demonstrate the presence of long-range ordered crystalline molecular packing. Under high-resolution TEM (HRTEM) analysis, the edges of micro-flowers (Figure S15d,e) expose the crystal lattice with a mean distance about 0.5 nm. Unlike uniform crystal lattice of most crystalline materials, the orientation of individual units roughly exhibits a helical arrangement, schematically

illustrated in Figure S15e. It was noted that the morphologies of self-assembled flowers are basically independent of water contents, but dependent on the C_{10}CN concentration. Scattered nanorods emerged instead of flower structures in the case of 2 mM concentration (Figures S15f and S19).

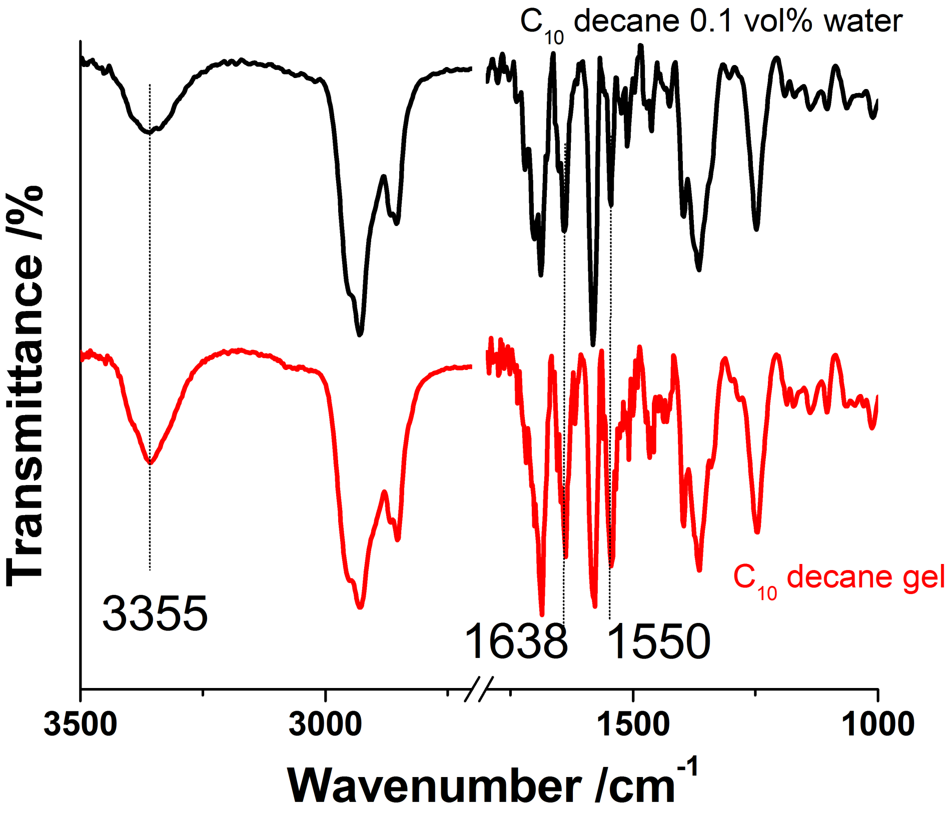

Figure S16 FT-IR spectra of gel sample and flower-like structure prepared from C_{10}CN. Peaks at 3355, 1638, and 1550 cm^{-1} are assigned to *N−H* stretch, amide-*I* stretch, and amide-*II* stretch bands respectively, which indicate the presence of inter-amide/imide hydrogen bonding interactions. Nevertheless, we noticed that the relative transmittance of amide-*I* stretch and amide-*II* stretch were reduced after the addition of water, elucidating that the inter-amide/imide hydrogen bonding was partially replaced by water.

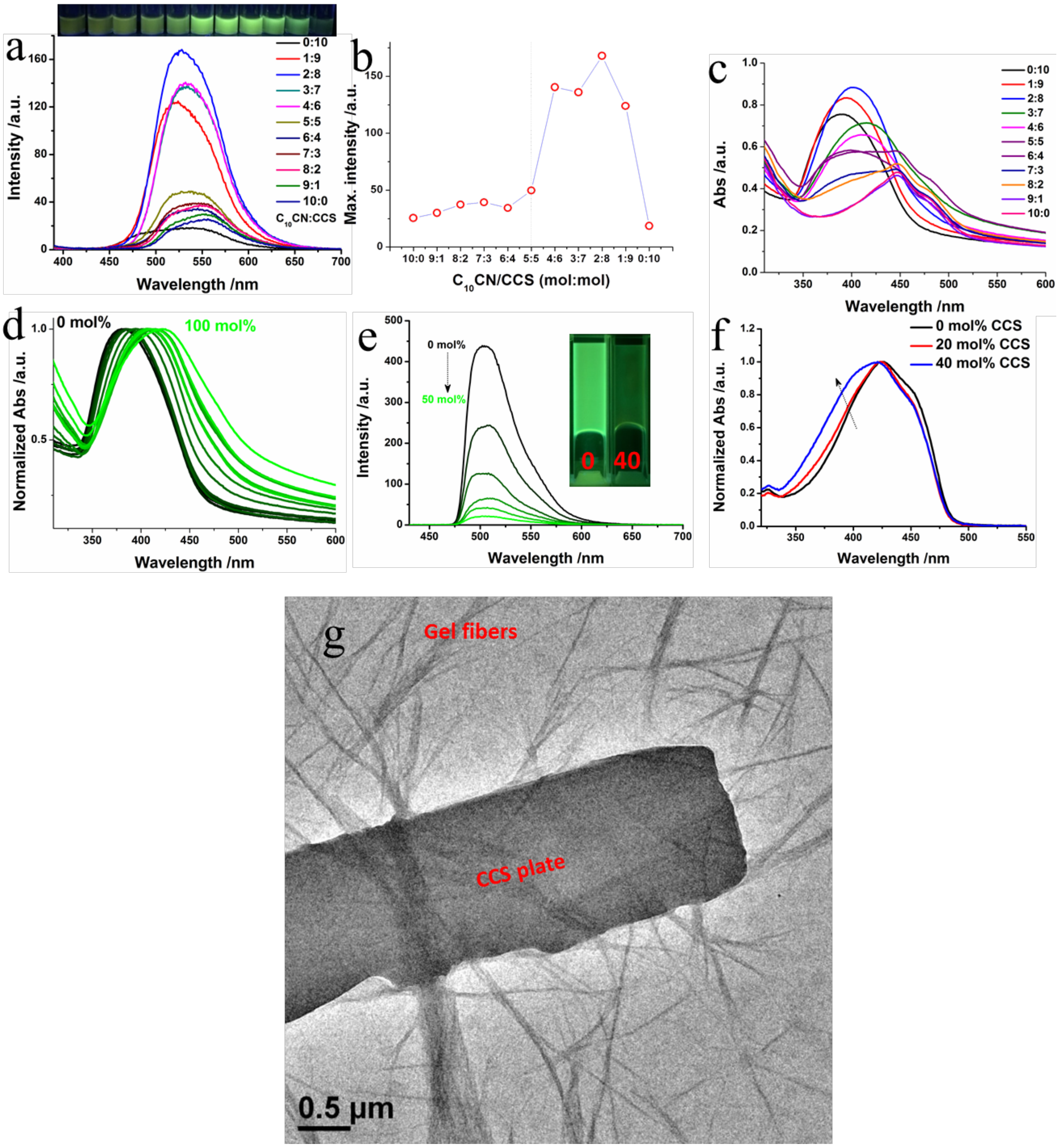

Figure S17 Molar ratio-dependent emission spectra (a,b) and UV-vis absorption spectra (b) of CCS/$C_{10}CN$ system, of which total concentration was fixed at 10^{-4} M. Inset of (a) shows digital images of co-assembly under 365 UV light, and the ratios of CCS and $C_{10}CN$ from left to right are 0:10, 1:9, 2:8, 3:7, 4:6, 5:5, 6:4, 7:3, 8:2, 9:1, and 10:0. UV-vis absorption of CCS (d) upon the titration of $C_{10}CN$ (concentration of CCS: 10^{-4} M). Fluorescent emission quenching upon the addition of CCS into $C_{10}CN$ decane gel (e), and the inset of which shows the emission comparison images of gels with (40 mol%) and without CCS. Normalized absorption with the addition of CCS. (g) TEM image of the self-sorted system of CCS/$C_{10}CN$ in decane (molar ratio of $C_{10}CN$ to CCS was 1:0.4).

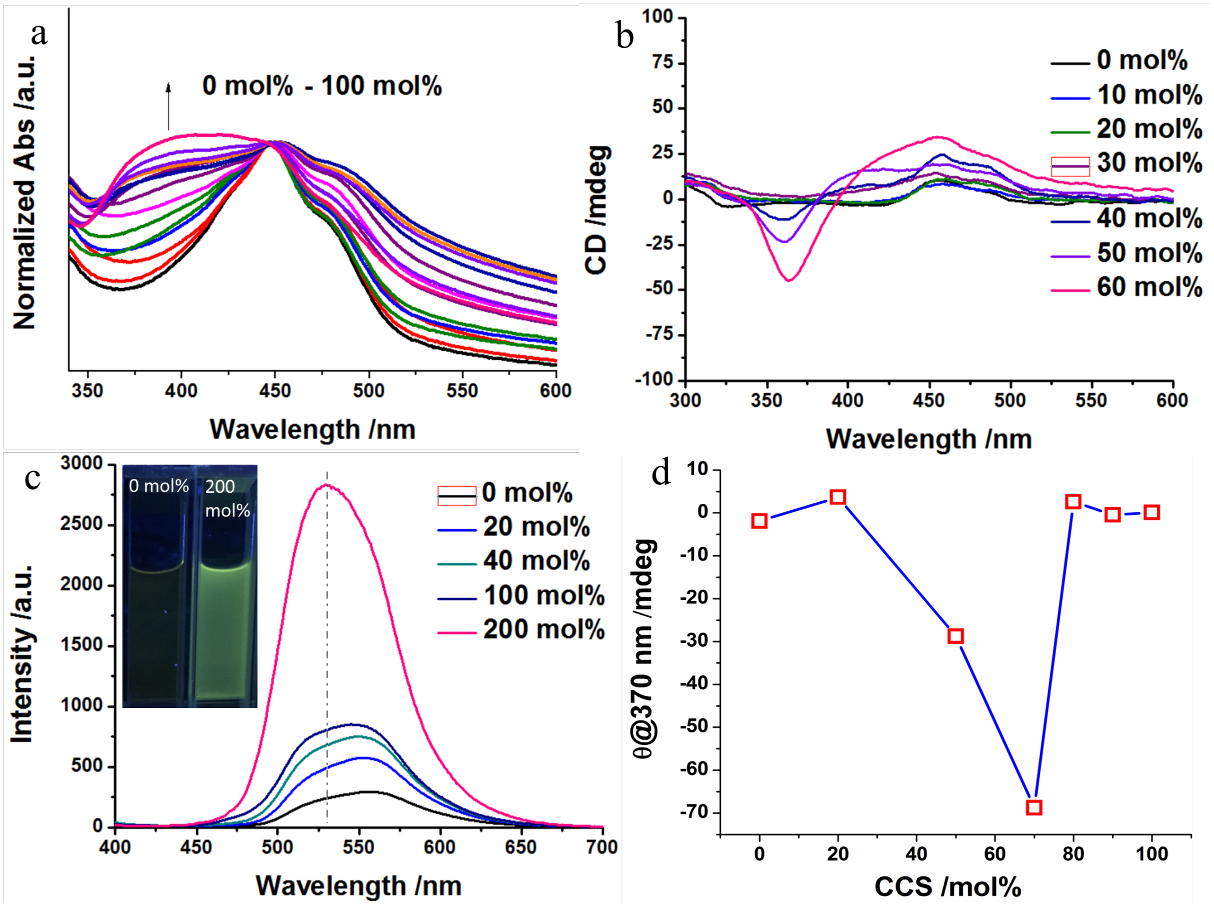

Figure S18 (a,b) Absorption and CD spectra by stepwise increasing the CCS component (from 0 mol% to 100 mol%) in $C_{10}CN$ self-assembled system (10^{-4} M, THF/water, 1/9, v/v). (c) Emission spectra of increasing CCS content in $C_{10}CN$ self-assembled system ($5x10^{-5}$ M, THF/water, 1/9, v/v). (d) CD values at 370 nm (absorption area of CCS) as a function of molar ratio of CCS and $C_{10}CN$ summarized from Fig. 5a.

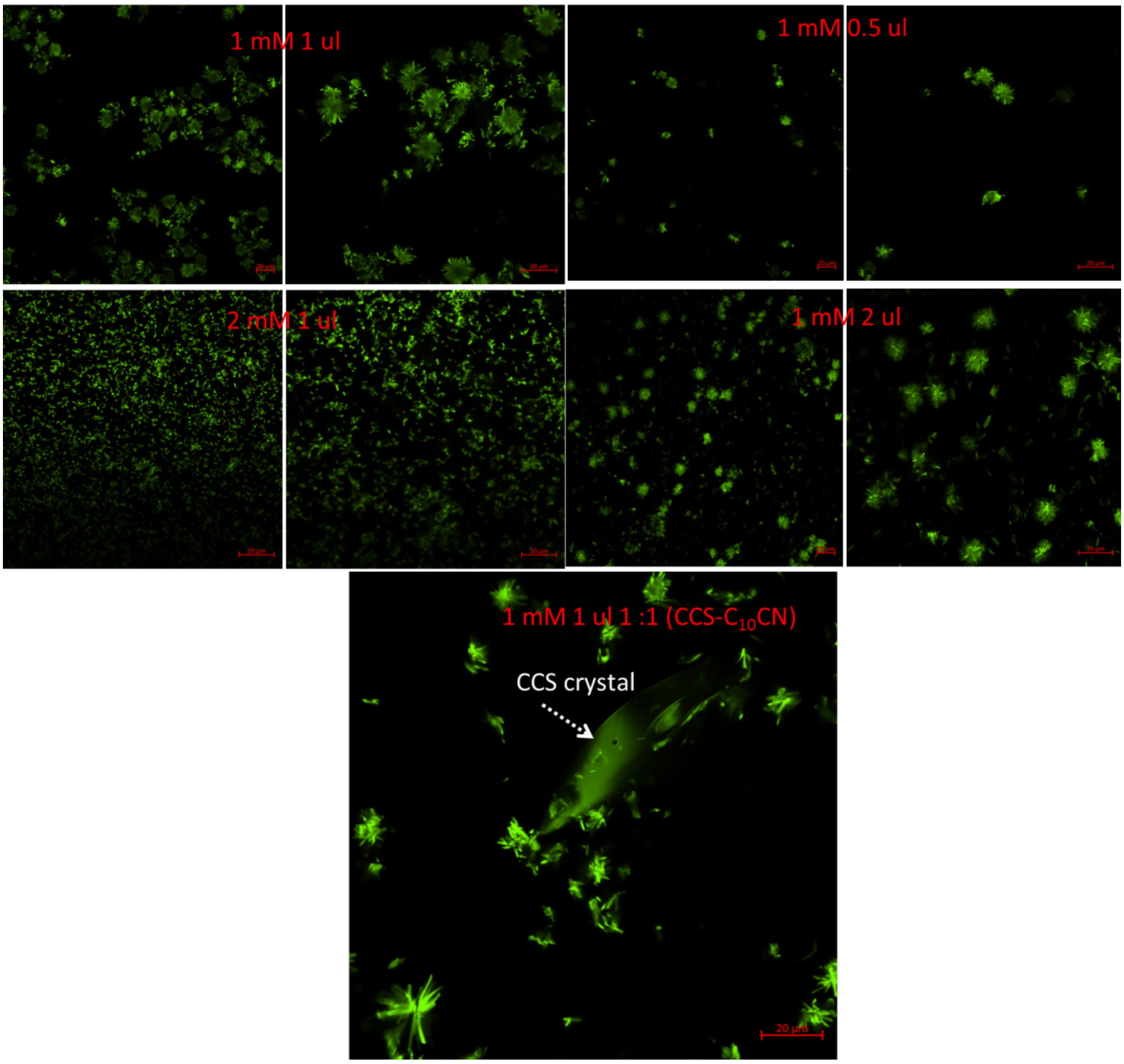

Figure S19 Water-induced microscale crystallization. CLSM images of samples with different concentrations and water fractions. All samples were prepared in THF-water mixture (1/9, v/v). The below image shows the water-induced crystallization in 1:1 mixture of CCS and $C_{10}CN$. The white arrow indicates the plate-shaped crystal of CCS in contrast to the flower-like $C_{10}CN$ crystalline objects, indicative of self-sorting in this solvent environment.

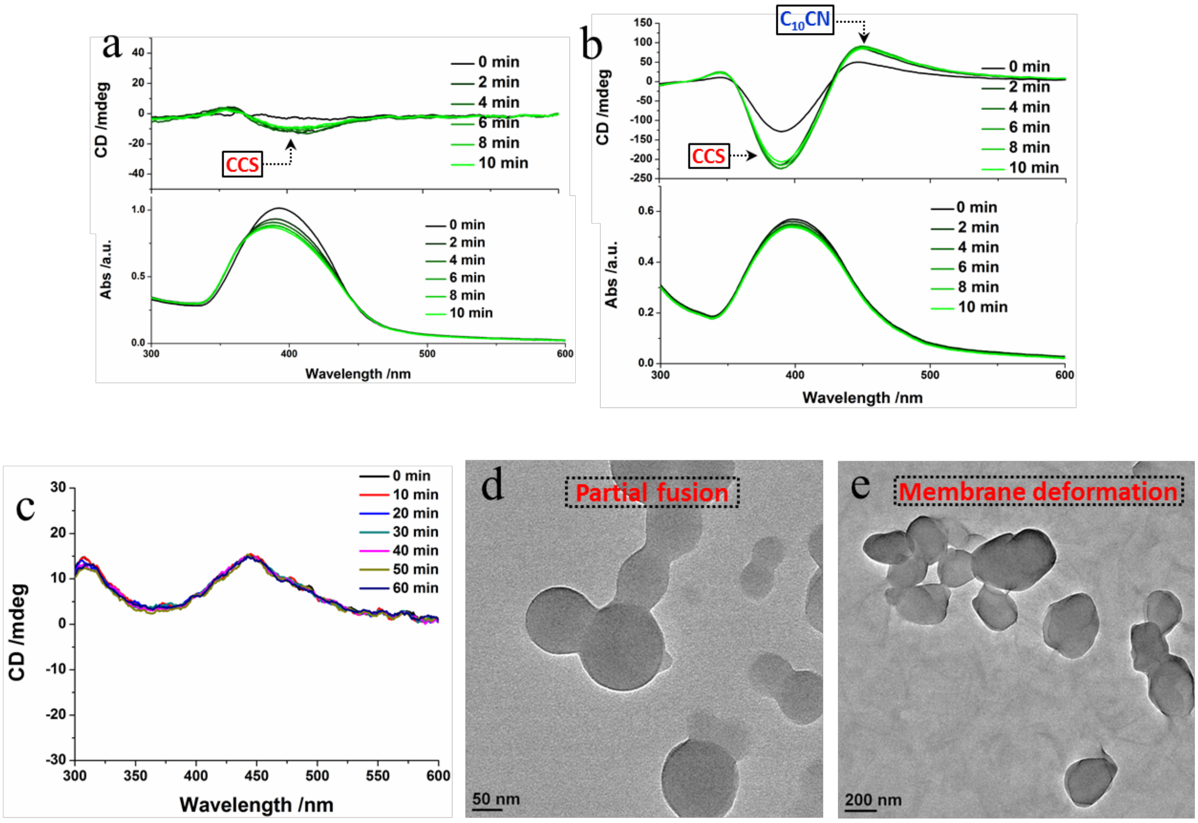

Figure S20 (a-c) CD and UV-vis spectra of samples with molar ratios of 9:0, 7:3, and 5:5 (CCS:C_{10}CN, total concentration: 10^{-4} M, f_w = 90 vol%) upon increasing the time of UV light irradiation (254 nm). (d,e) TEM images of samples with molar ratios of 5:5 and 2:8 (CCS:C_{10}CN, total concentration: 10^{-4} M) under sufficient light irradiation.

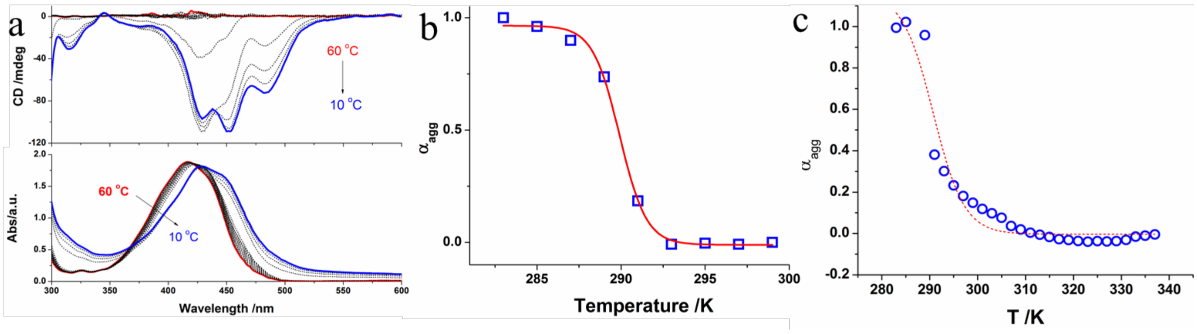

Figure S21 (a) Temperature-dependent UV-vis and CD spectral changes of C_{10}CN in decane (2×10^{-4} M, 2 °C interval). (b) Degree of aggregation as the function of temperature. (c) Aggregation degree as a function of temperature, calculated from temperature-dependent UV-vis absorption profile. Red line: Boltzmann fit.

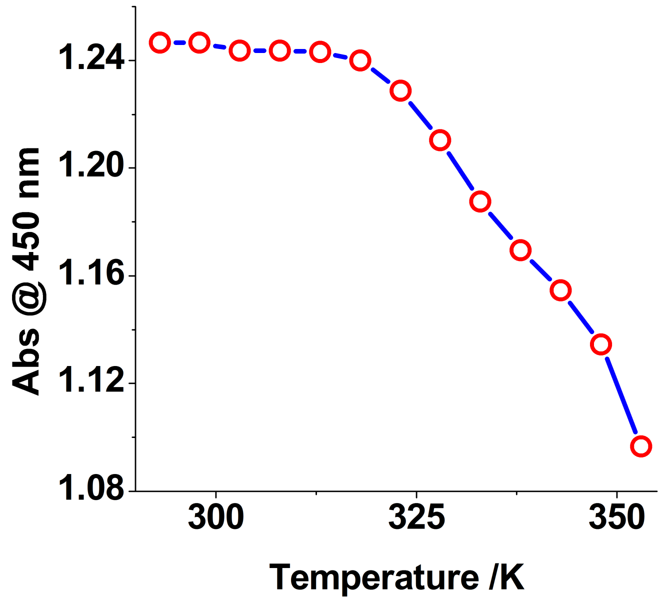

Figure S22 Absorbance variation of $C_{10}CN$ vesicle system (10^{-4} M, f_w = 90 vol%) at 450 nm with the increase of temperature.

Table S1 Crystallographic data for $C_{10}CN$

Formula	C_{54} H_{85} N_3 O_6
Mr	807.6
Temperature (K)	293
Wavelength	1.54178 Å
Crystal system	triclinic
Space group	P 1
a, b, c/ Å	a 9.8067(4) b 11.3451(4) c 23.8012(12)
V, $Å^3$	2606.75
Cell angles	α 95.974(4) β 91.749(4) γ 97.784(3)
Z, Z'	2, 0
R-factor (%)	6.33

Table S2 Crystallographic data for CCS

Formula	C_{48} H_{67} N_3 O_3
Mr	733.5
Temperature (K)	293
Wavelength	1.54178 Å
Crystal system	Monoclinic
Space group	C 2
a, b, c/ Å	a 51.912(3) b 7.5465(4) c 23.4693(8)
V, $Å^3$	8658.27
Cell angles	α 90(4) β109.659(4) γ 90
Z, Z'	8, 0
R-factor (%)	8.74

References

S1. P. Xing, H. Chen, L. Bai, A. Hao, Y. Zhao, *ACS Nano* **2016**, *10*, 2716-2727.

S2. P. Xing, H. Chen, L. Bai, Y. Zhao, *Chem. Commun*. **2015**, *51*, 9309-9312.
